# Thermal and structural studies of carbon coated Mo_2_C synthesized via *in-situ* single step reduction-carburization

**DOI:** 10.1038/s41598-017-03197-8

**Published:** 2017-06-14

**Authors:** Rameez Ahmad Mir, Piyush Sharma, Om Prakash Pandey

**Affiliations:** School of Physics and Materials Science, Thapar University, Patiala, 147 004 India

## Abstract

Carbon coated nano molybdenum carbide (Mo_2_C) has been synthesized at 800 °C through single step reduction route using molybdenum trioxide (MoO_3_) as a precursor, polypropylene (P.P) as a carbon source and magnesium (Mg) as a catalyst in an autoclave. The synthesized samples were characterized by X-ray diffraction (XRD), thermal analysis techniques (TG/DTA/DTG), field emission scanning electron microscopy (FESEM) and transmission electron microscopy (TEM). Williamson- Hall (W-H) analysis has been done to estimate various parameters like strain, stress and strain energy density. Multi-stage kinetic analysis of the product phase has been studied to establish the nature of the thermal decomposition. Coats-Redfern method applied to determine the mechanism involved in the decomposition of the product phase shows that initial and final stage follow F1 mechanism whereas middle stage follow F3 mechanism. The activation energy (*E*_a_) and pre-exponential factor (*A*) has also been determined. The morphological studies shows that the particles have partially spherical/faceted shape, with carbon coated having wide particle size distribution. The surface chemistry and surface area analysis were studied by X-ray photoelectron spectroscopy (XPS) and Brunauer-Emmet-Teller (BET), respectively. The formation mechanism of carbon coated Mo_2_C nano particles has been predicted based on the XRD, TG/DTA & DTG and microstructural results.

## Introduction

The industrial demand of transition metal carbides (TMCs) is increasing due to their remarkable physical and chemical properties. The high melting point, good conductivity, thermal stability, excellent corrosion, wear resistance and catalytic properties similar to noble metals of Mo_2_C find many industrial applications^1–5^. The Mo_2_C particles are highly active HER (Hydrogen Evolution Reaction) catalyst and has stability in both acidic and basic mediums^6, 7^. Because of these extensive applications of Mo_2_C, synthesis of nanocrystalline Mo_2_C utilizing low cost carbon source by simple route at lower temperatures is eminently desirable. The catalytic activity of Mo_2_C is mostly influenced by surface structure and elemental composition, which are dependent on the synthesis route^8^.

Traditionally, the micron size Mo_2_C is produced by direct carburization of molybdenum and molybdenum oxide powders at higher temperatures^9^. However, for the synthesis of nano Mo_2_C powder different procedures are adopted^10–17^. The structure and crystallite size of carbides mainly depends on the synthesis temperature, type and concentration of carbon source^18–20^. The morphology of particles depends upon nature of the carbon and reaction time that plays an important role. Chen *et al*.^21^ synthesized Mo_2_C at 600 °C using MoO_3_ in an autoclave in presence of  Mg and CH_3_COOK as reducing agent and carbon source, respectively. However, the authors did not establish the mechanism of reduction. Moreover, the processing parameters have not been optimized.

In this paper, synthesis of Mo_2_C through a simple reduction and carburization of MoO_3_ in an autoclave is reported. For the synthesis, polypropylene and Mg are used as carbon source and catalyst, respectively. Polypropylene is a thermoplastic polymer and used for manufacturing variety of plastics. In order to recycle the plastics and to conserve the natural products, the current path is followed^22^. Furthermore, the kinetic analysis involved in the thermal decomposition process is crucial to understand the thermal stability of materials for wide range of applications. Numerous researchers have evaluated various kinetic parameters (activation energy, pre-exponential factor and co-relation factor) and proposed the reaction mechanism, by adopting well known thermal kinetic models^23–27^. However, so far no kinetic study has been done to determine the kinetic parameters and reaction mechanism involved in the thermal decomposition of Mo_2_C. Coats-Redfern method has been utilized to determine the mechanism and kinetic parameters involved in the thermal decomposition of the synthesized sample^28^. The thermodynamic parameters considering the possible decomposition route of polypropylene has been investigated to study the reduction-carburization mechanism.

## Results and Discussions

### X-ray diffraction analysis of synthesized powders

**Figure 1:**
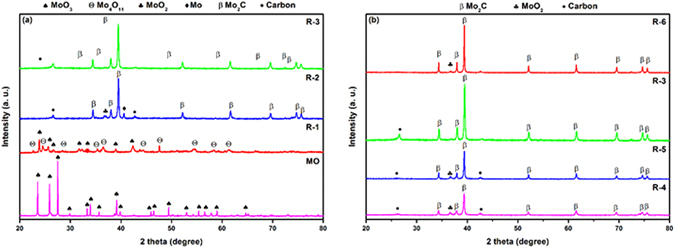
(**a**) XRD pattern of MO and samples synthesized at different temperatures: 600 °C (R-1), 700 °C (R-2), 800 °C (R-3) with constant holding time of 10 h. (**b**) XRD pattern of samples synthesized at 800 °C with different holding time: 1 h (R-4), 2 h (R-5), 10 h (R-3) and 12 h (R-6).

Table 1 gives the details of reaction parameters and the percentage compositions of major and minor phases for samples (R-2 to R-6). The XRD pattern of sample (R-1) synthesized at 600 °C for 10 h reveals the presence of MoO_3_ (ICDD reference -089-7112) and minor intermediate phase Mo_4_O_11_ (ICDD reference -072-0447) as shown in Fig. 1a, indicating that the temperature is not sufficient enough for the carbon diffusion and to reduce the MoO_3_ to MoO_2_^29^. Moreover, the reduction of MoO_3_ to MoO_2_ in presence of hydrogen and carbon proceed via formation of an intermediate phase Mo_4_O_11_^30^.

**Table 1:** Synthesis parameters of reaction and % age of phase compositions in samples (R-2) to (R-6). Williamson-Hall analysis and Scherrer criterion of samples (R-2 to R-6).

Sample ID	Temperature (°C)	Soaking time (hrs.)	Phase compositions (%)				UDM		Scherrer
			Mo_2_C	C	MoO_2_	Mo	D (nm)	ε × 10^−4^	D (nm)
R-1	600	10	MoO_3_, Mo_4_O_11_,C				—	—	—
R-2	700	10	86.8	5.2	3.8	4.2	58.01	7.848	38.00
R-3	800	10	96.0	4.0	0.0	0.0	47.32	5.858	36.36
R-4	800	2	88.2	5.6	6.2	0.0	38.19	4.189	31.63
R-5	800	5	90.8	5.2	4.0	0.0	53.96	4.370	41.70
R-6	800	12	96.5	2.3	1.2	0.0	96.29	4.282	63.16

With the increase in temperature upto 700 °C (R-2), XRD pattern shows an increase in the peak intensity of Mo_2_C (ICDD reference -035-0787) phase with decreasing minor phases (Table 1), indicating that the reaction temperature is still not sufficient for carbon diffusion to carburize the reduced oxide phase MoO_2_ (ICDD reference -078-1070) and molybdenum (Mo) (ICDD reference -089-5156) to pure Mo_2_C. The reduction-carburization of MoO_3_ to Mo_2_C proceeds via two steps as given in equations, S1.1 and S1.2 Supplementary Information (SI) ^31^. R-2 sample shows the feasibility of reaction path S1.1 (SI). However, the conversion of MoO_3_ to Mo_2_C is accomplished at 800 °C (R-3). All peaks correspond to Mo_2_C with no minor phase except single peak of excess carbon (ICDD reference-026–1076) as shown in Fig. 1a, confirming the complete diffusion of carbon to form pure Mo_2_C and the most favorable reaction path as given in S1.2 (SI).

**Figure 2:**
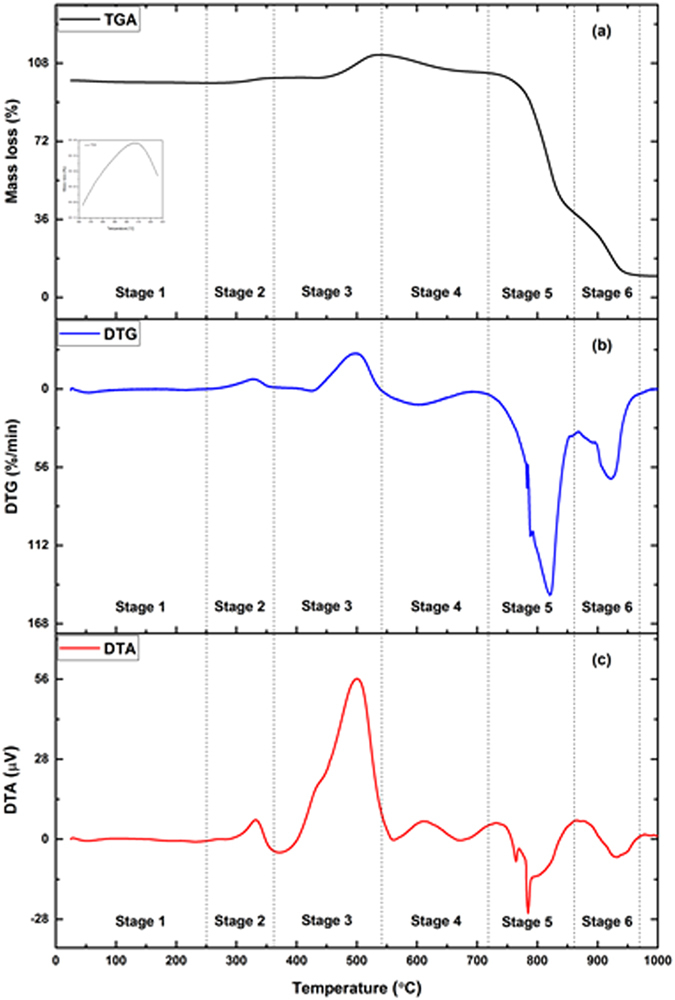
Thermal behaviour of sample R-3, (**a**) TGA, (**b**) DTG, (**c**) DTA.

In order to reduce the soaking time for R-3 (800 °C, 10 h), experiments employing different soaking times have been done (Table 1). Figure 2b shows the XRD pattern of samples synthesized at 800 °C for different soaking times. With the increase in soaking time up to 10 h, the samples show an ascending peak intensity of Mo_2_C with diminishing tendency of minor phase (Table 1). The results confirm that diffusion of carbon into MoO_3−x_ (x = 0,1) increases with increase in the time. This confirms that the reaction time of 10 h at 800 °C is sufficient enough for reduction and carburization of MoO_3_ to Mo_2_C. It also shows the feasibility of most favorable reaction (equation S1.2, SI) for solid-state reactions. Moreover, further increase in soaking time to 12 h in R-6 sample, the minor phase of MoO_2_ appears because of decarburization and oxidation. At higher temperature, affinity of oxygen diffusion is more when the reaction time is sufficient enough for oxygen to diffuse into Mo_2_C to favor reversible reaction^29, 32^. The carburizing atmosphere inside the autoclave is very different involving the interaction of numerous gases. The methane and other hydrocarbon gases like ethane, propane, ethylene and propylene etc. generated by decomposition of polypropylene may break into C and H_2_ and facilitate the carburization more rapidly^33, 34^. In contrast to this CO_2_ and H_2_O being byproducts of decomposition reactions act as decarburizing agents. However, certain amount of CO_2_ is tolerable at defined carburizing temperature, without causing decarburization action. Presence of CO_2_ in small amount requires a high concentration of CO to balance the decarburization action^35^.

The XRD results of R-3 confirm the formation of pure phase Mo_2_C. The data was fitted via Rietveld refinement for hexagonal space group P63/mmc (194) to confirm the crystal structure. Figure S1a (SI) shows the Rietveld refinement of the product. The Wyckoff positions used for Rietveld refinement of XRD data are 2(c) 1/3, 2/3, 1/4; 2/3, 1/3, 3/4 for Mo and 2(a) 0, 0, 0; 0, 0, 1/2 for C^36^. The R_wp_ and χ^2^ values obtained from the Rietveld refinement are 6.92 and 1.95, respectively.

### Crystallite Size and Strain

The peak position, 2θ (Bragg Peaks) and full width half maxima β_observed_ of peaks were calculated by X-ray line profile fitting technique using the Gaussian function. The result of curve fit for peak (100) is shown in Fig. S1b (SI). The breadth of the Bragg peaks combines both instrumental and sample dependent effects. The instrumental corrected broadening^37^ *β* corresponding to diffraction peak of Mo_2_C was calculated using following relation:

1 $${{\boldsymbol{\beta }}}^{2}=[{({{\boldsymbol{\beta }}}^{2})}_{{\boldsymbol{Observed}}}\,-\,{({{\boldsymbol{\beta }}}^{2})}_{{\boldsymbol{Instrumental}}}]$$

Using Scherrer equation^38^, Scherrer plot was drawn. By fitting the data, crystallite size D was calculated from slope of the fit line as shown in Fig. S1c (SI). Peak broadening in above case is attributed coherently due to the crystallite size.

### Williamson-Hall methods

Williamson-Hall analysis Uniform deformation model (UDM),(S1.3, SI), uniform stress deformation model (USDM) and uniform deformation energy density model (USEDM) with details and equations in appendix S1 (SI) has been done. The slope and intercept of graph plotted between ‘β Cosθ’ as function of ‘4 Sinθ’, measures the magnitude of micro strain ε and crystallite size, respectively as shown in Fig. S1d (SI). The positive value of strain confirms the compression in lattice.

The UDM model in Fig. S1d (SI) proves to be more appropriate with less scattering of data away from the linear expression as compared to USDM and USEDM as shown in Fig. S1e,f (SI), respectively. The decrement in the value of strain with rise in temperature at constant synthesis time R-2 to R-3 has been observed and is listed in Table 1 and Table S1 (SI), respectively. This may be due to the accomplishment of reaction process, where maximum pure phase formation of Mo_2_C takes place and the system attains more stable state. The increment in value of the strain from reaction time 2 h to 10 h at 800 °C shows that the reaction path is in the forward direction. It confirms that the synthesized powder will have higher value of strain with the movement of reaction in forward direction to form Mo_2_C particles, which decreases when there is reversibility of the reaction (R-6). The positive value of strain determines that the sample undergoes a tension state.

### Thermal Analysis

Thermal behavior of best sample R-3 is investigated through a group of thermal analysis techniques. TGA, DTG and DTA curves from room temperature to 1000 °C in air is shown in Fig. 2a–c. This figure clearly indicates that the thermal decomposition of the prepared sample occurred in six stages including peaks of mass loss and mass gain. The oxidation of carbonaceous residue shows the sign of mass loss while the oxidation of Mo_2_C species show mass gain. In stage 1, the observed mass loss of ~1% (Fig. 2a) occurred due to the removal of moisture and other volatile species adsorbed on the sample surface^39^. In stage 2, the mass gain observed is ~2.3% at ~326 °C (Fig. 2a). This may be related to the oxidation of undetermined Mo metal in XRD pattern and meanwhile the oxidation of Mo_2_C started, which led to the formation of intermediate phases of molybdenum oxide. Also, the reaction is found to be exothermic in nature as indicated by DTA (Fig. 2c) peak at ~330 °C.

In stage 3, the mass remains almost constant upto 400 °C (Fig. 2a) due to the presence of excess carbon on the outer layer of the sample causing hindrance in oxidation of Mo_2_C. As temperature increases, the outer layer of carbon gets oxidized and oxidation of Mo_2_C occurs. So, there is a slight mass loss in temperature range 400 °C to 430 °C, shown in the inset of Fig. 2a. The prominent mass gain and sharp exothermic peak (Fig. 2b,c) at 500 °C may be due to the transformation of Mo_2_C to MoO_x_ (2<x ≤ 3). The thermal stability of R-3 sample is estimated by mass gain (Fig. 2a) occurring due to oxidation of Mo_2_C specie^40^. Afterwards, the decomposition of R-3 sample shows mass loss (Fig. 2a) of ~9% in stage 4. This mass loss is associated with the combustion of carbon residue. The oxidation of intermediate oxides having stoichiometry close to MoO_3_ gets transformed to more stable phase MoO_3_. In this temperature range, carbon residue hinders further oxidation of Mo_2_C but itself gets oxidized to CO_2_. The remained Mo_2_C is encapsulated inside MoO_3_ layer formed during oxidation. Also, DTG and DTA curves (Fig. 2b,c) of this stage shows that the decomposition is exothermic in nature having broad exothermic and mass loss peak at ~600 °C. Khilborg *et al*.^41^ defined the temperature range for transformation of various intermediate molybdenum oxides. They studied the transformation from MoO_2_ to MoO_3_ and suggested that the intermediate phase Mo_17_O_47_ forms at about 560 °C which converts into Mo_4_O_11_ at 630 °C. In addition, Lopez *et al*.^42^ determined the phase transformation of MoO_2_ to MoO_3_ via intermediate oxides and predicted that the mixture of both MoO_2_ and MoO_3−x_ are present in the temperature range 300–600 °C. The monoclinic MoO_3−x_ is transformed to more stable orthorhombic MoO_3−x_ species at 440–570 °C.

In stage 5, intermediate Mo oxides thoroughly transformed into stable orthorhombic MoO_3_, which melts around 800 °C. A rapid mass loss of ~65% is shown in TG curve (Fig. 2a), which correspond to the sublimation of MoO_3_ at higher temperatures (above 740 °C) as predicted by Chen *et al*.^43^. In this stage, decomposition of molybdenum oxide occurred in steps as observed from DTG (Fig. 2b). DTG curve exhibit a shoulder peak at ~790 °C along with prominent peak of mass loss at ~820 °C. The sharp endothermic peaks observed in DTA (Fig. 2c) may be associated with the melting of mixed oxide phases of Mo (765 °C) and MoO_3_ (785 °C). It may be due to the decomposition of intermediate molybdenum oxide species, which readily get transformed to MoO_3_ and melted at higher temperatures. Molybdenum exists in different oxidation states and has variety of oxides and sub oxides. Mo (IV) and Mo (VI) oxide species are the only stable phases at high temperatures. Kihlborg *et al*.^44^ reported that Mo_18_O_52_, an intermediate phase forms in a temperature range 600–750 °C and transforms to more stable phase of MoO_3_. Furthermore, another intermediate phase Mo_8_O_23_ forms in temperature range 650–780 °C and transforms to Mo_9_O_26_ at ~780 °C. These phases get readily decomposed to MoO_3_ at 800 °C, which evaporates instantaneously. In addition, the orthorhombic phase Mo_4_O_11_ having stability greater than 800 °C in certain cases may transform to MoO_2_ and MoO_3_ above 850 °C or may have formed due to the comproportionation of MoO_3_ and MoO_2_. However, it is difficult to establish the time duration and temperature range for the stability of these intermediate oxide phases as they get readily transformed from one intermediate phase to another one. Furthermore, the transformation observed in stage 6 is associated with oxidation of MoO_2_ and MoO_3_ mixed phases, which were formed due to the decomposition of orthorhombic phase Mo_4_O_11_. The maximum mass loss peak at ~925 °C and endothermic peak at ~930 °C is observed from DTG and DTA curves (Fig. 2b,c), respectively. Moreover, TG curve shows stability in mass at temperature above ~940 °C corresponding to MoO_2_ phase retained at higher temperatures.

### Multi-stage kinetic analysis

The data obtained from thermographs was used to evaluate kinetic parameters and reaction mechanism involved in thermal decomposition of the sample. In this study, Coats-Redfern kinetic model was employed to determine various thermal kinetic parameters and reaction mechanism^28^. In order to identify the exact reaction mechanism, the linear fitted curves corresponding to various reaction mechanisms Table S2 (SI) for each stage of sample decomposition, are shown in Fig. S2. (1–5), SI. Moreover, for linear fitting of data, experimental points based on degree of conversion (*α*) were selected from 0–1 with the interval of 0.01. The reaction mechanism showing good correlation factor (≈1) was considered to be exact mechanism involved in the thermal decomposition process. Also, thermal decomposition of sample occurred in six stages, so it is crucial to conduct a multi-stage kinetic analysis. Since in stage 1, the mass loss is very less (<1%), so it is not considered for kinetic analysis.

**Figure 3:**
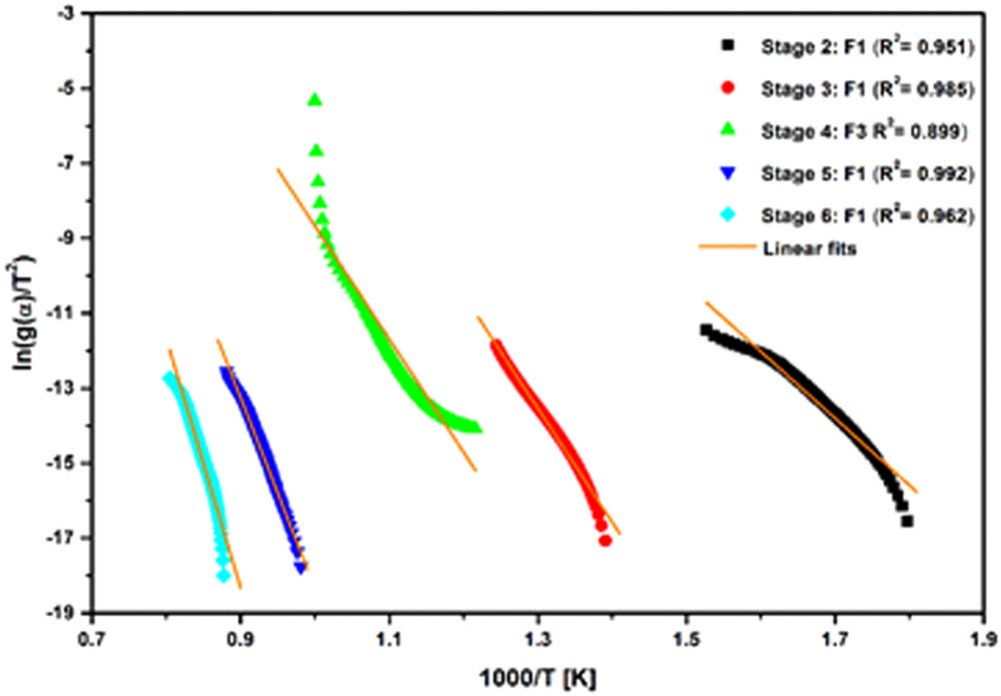
Best linear fitted curves corresponding to Random nucleation method in stage 2, 3, 5, 6 and Nucleation and growth mechanism in stage 4.

The best linear fitted curves of reaction mechanism for stage 2–6 are illustrated in Fig. 3. In stage 2 (Fig. S2.1b, SI) the best correlation factor (R^2^ = 0.951) was obtained in F1 random nucleation mechanism. However, all other mechanism such as diffusion, nucleation and growth and phase boundary controlled (Fig. S2.1a,c & d, SI) mechanism showed poor value of correlation factor as compared to F1 mechanism. This implies that the thermal decomposition process followed F1 mechanism in stage 2. After identification of reaction mechanism, kinetic parameters viz. activation energy (E_a_) and pre-exponential factor (A) along with standard deviation (SD) were determined and presented in Table 2. The value of activation energy and pre-exponential factor was found to be 148.27 kJ mol^−1^ and 1.33 × 10^9^ min^−1^, respectively. In the same way, reaction mechanism and kinetic parameters were determined for all stages of decomposition. In stage 3 (Fig. S2.2b, SI), the reaction mechanism remains to be F1 with correlation factor of 0.985. These results indicated that the thermal decomposition process followed F1 mechanism during mass gain due to oxidation of the sample in both stages. The activation energy calculated for stage 3 is 252.33 kJ mol^−1^, which is higher in comparison to stage 2. The increment in activation energy is related to the hindrance offered by the outer layer of carbon, in order to oxidize Mo_2_C. The calculated value of pre-exponential factor is 2.74 × 10^13^ min^−1^. As the temperature increased, in stage 4 (Fig. S2.3b, SI) the reaction mechanism changed from F1 to F3 with the correlation factor of 0.899. This change in mechanism may be associated with mass loss during simultaneous combustion of carbon residue and oxidation of intermediate molybdenum oxides. In this stage the activation energy slightly drops to 250.87 kJ mol^−1^ and the pre-exponential factor is found to be 3.3 × 10^11^ min^−1^.

**Table 2:** Calculated value of activation energy (E_a_) and pre-exponential factor (A) along with standard deviation for each stage of decomposition.

Stages	Reaction Mechanism	Activation Energy (KJ mol^−1^)	Standard deviation in activation energy	Pre-exponential factor (min^−1^)	Standard deviation in pre-exponential factor
Stage 1	F1	148.27	34.073	1.33 × 10^9^	6.858
Stage 2	F1	252.33	31.115	2.74 × 10^13^	4.899
Stage 3	F3	250.87	84.895	3.3 × 10^11^	11.457
Stage 4	F1	422.81	39.204	3.36 × 10^16^	4.359
Stage 5	F1	552.55	111.052	3.47 × 10^20^	11.289

**Figure 4:**
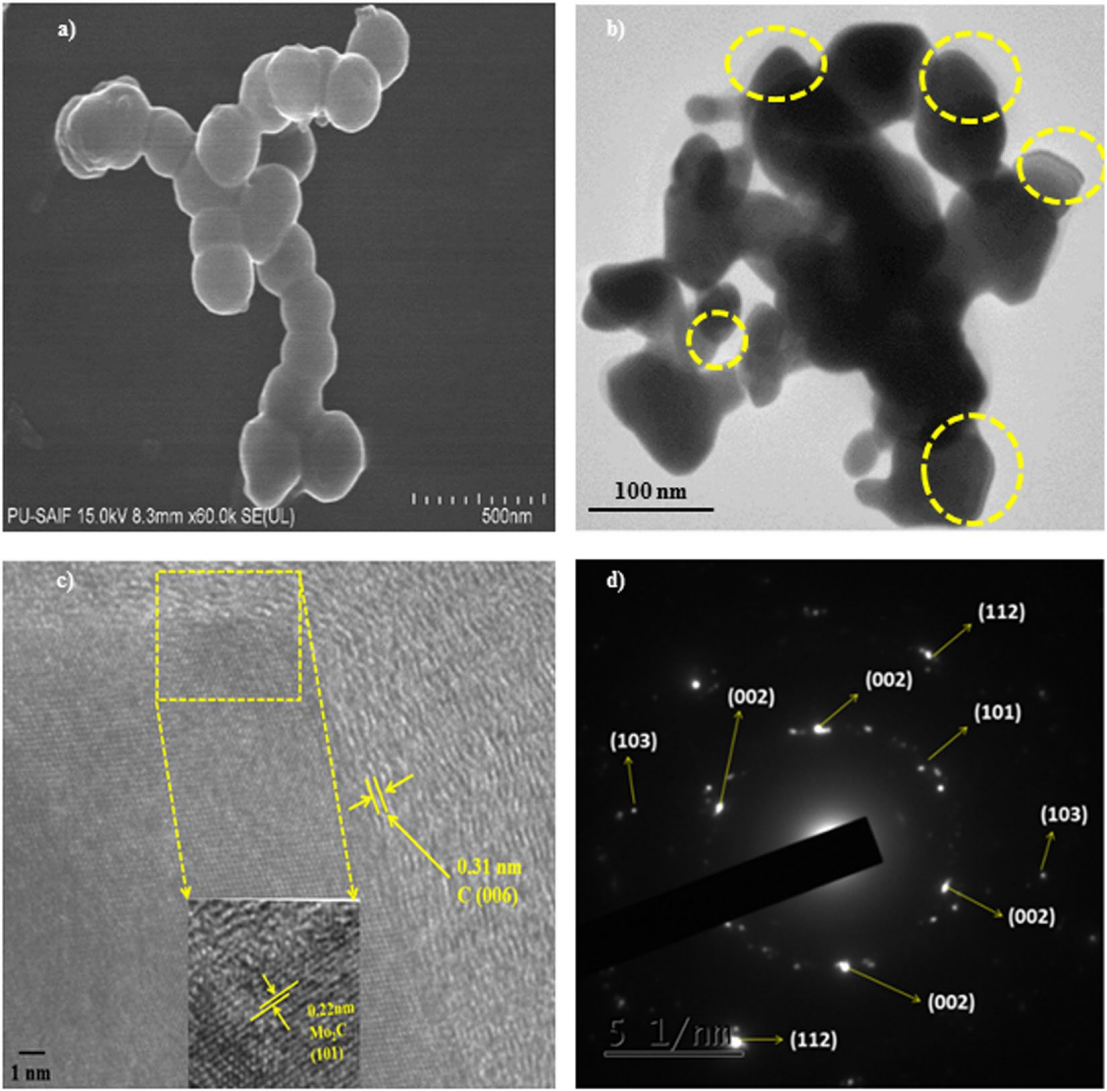
(**a**) FESEM micrograp of Mo_2_C nanopowder R-3 sample showing spherical/faceted morphology. (**b**) TEM micrographs of Mo_2_C (R-3 sample). (**c**) HRTEM micrograph of Mo_2_C nnaopowder, R-3 sample showing lattice fringing corresponding to (101) plane and (**d**) SAED pattern.

Furthermore, the reaction mechanism again shifted to F1 random nucleation mechanism in stage 5 (Fig. S2.4b, SI). The observed value of correlation factor for F1 mechanism is 0.992. The activation energy increased abruptly to 422.81 kJ mol^−1^, which may correspond to sharp mass loss due to the melting of mixed Mo oxide phases and MoO_3_. Also, the value of pre-exponential factor increased to 3.36 × 10^16^ min^−1^. Similarly, stage 6 (Fig. S2.5b, SI) demonstrated that F1 mechanism is followed by thermal the decomposition process. Figure 4e shows that F1 mechanism exhibit better value of correlation factor (R^2^ = 0.962) as compared to other mechanism (Fig. S2.5). In this stage, the activation energy and pre-exponential factor is 552.5 kJ mol^−1^ and 3.47 × 10^20^ min^−1^ respectively. The increment in kinetic parameters is due to the formation of stable mixed phase of MoO_2_–MoO_3_, which further oxidized into highly stable phase MoO_2_.

### Microstructural Analysis

Figure 4a and Fig. S3 shows the FE-SEM images of the sample (R-3) synthesized at 800 °C for 10 h. Figure 4a and Fig. S3 clearly depicts that the particles have tendency to agglomerate and particle size distribution is inhomogeneous. The morphological analysis of the particles in FE-SEM shows that the agglomerated particles have spherical to faceted morphology and the size varies from 65–150 nm.

Figure 4b shows the TEM images of sample (R-3) synthesized at 800 °C for 10 h. The clear visibility of surface coating of particle confirms the presence of carbon (circled). The carbon coated Mo_2_C particles are embedded in the carbon matrix and have tendency to agglomerate. Figure 4c shows the High Resolution-TEM (HRTEM) images of carbon coated Mo_2_C and the lattice fringes of Mo_2_C in HRTEM micrograph correspond to (101) plane of hexagonal Mo_2_C (ICDD pattern 035–0787) and the inter planar distance is 0.22 nm. The SAED pattern (Fig. 4d) identifies the different planes of Mo_2_C. The micrograph clearly indicates the polycrystalline nature of the synthesized sample.

The particle distribution size observed from TEM images is broad (Fig. S3.1, SI). Nearly about 100 particles from different areas/scans were measured and the particle size follows log-normal distribution. The particle size obtained from TEM is comparable to that obtained from XRD analysis. However, certain deviation in size using different methods may be due to the agglomeration of particles^20^.

### X-ray photoelectron spectroscopy (XPS) analysis

**Figure 5:**
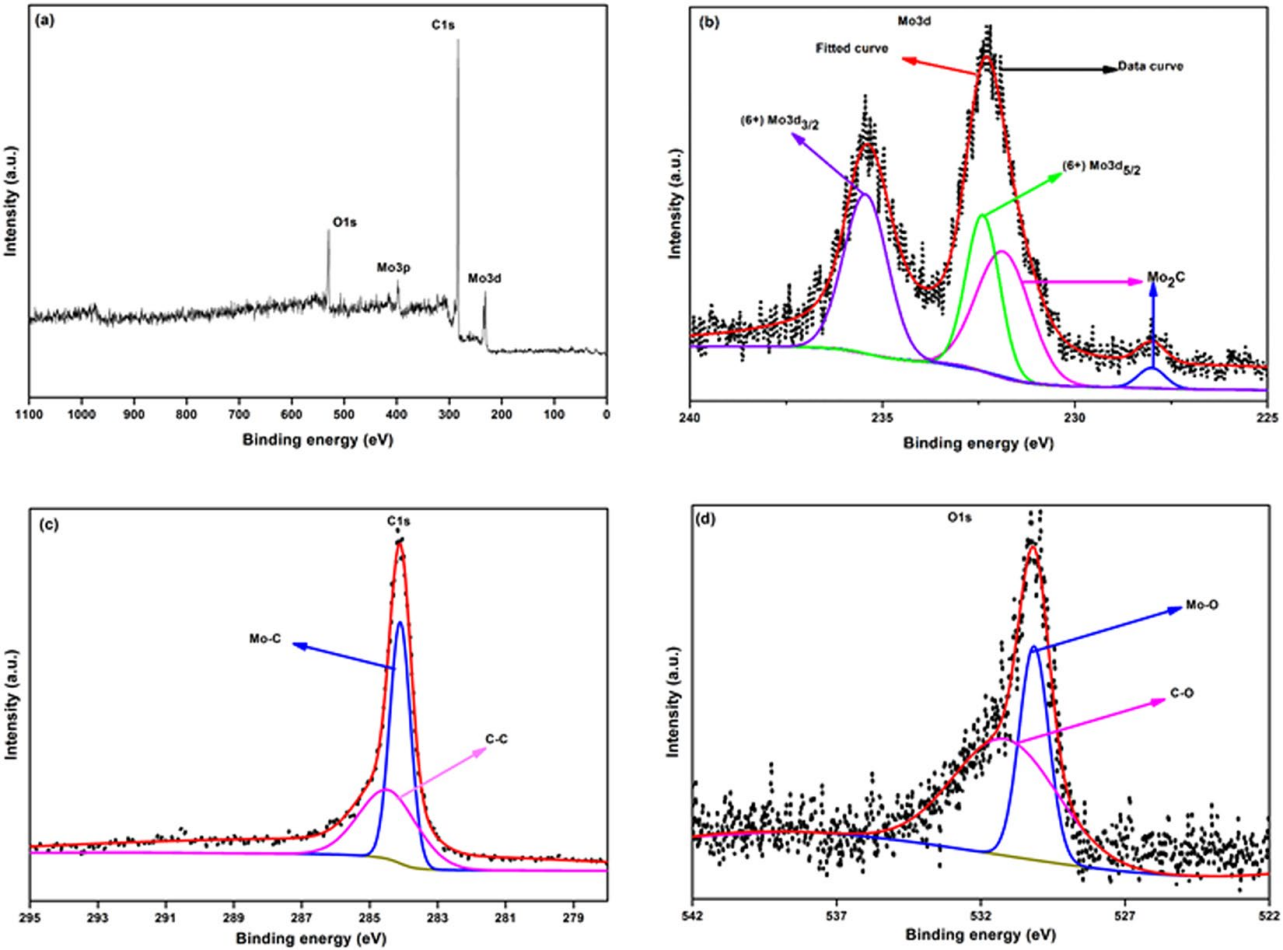
XPS spectrum of R-3 sample (**a**) full survey (**b**) Mo3d (**c**) C1s and (**d**) O1s spectrum.

The composition and surface chemical state of carburized products R-3 and R-6 samples were further identified by X-ray photoelectron spectroscopy with peak deconvolution. Figure 5a shows the full survey XPS spectrum of R-3 sample. The survey spectrum shows the presence of five distinct peaks at 232.9, 284.8, 397.7 532.4 eV, which can be attributed to Mo3d, C1s, Mo3p, and O1s respectively^45^. By means of curve fitting, the distribution of molybdenum (Mo) species and the corresponding oxidation states were estimated. The high resolution (HR) spectrum of Mo3d is shown in Fig. 5b, which reveals two peaks at 228.1 and 231.7 eV assigned to (Mo^2+^) Mo 3d_5/2_ and Mo3d_3/2_ respectively, originating from Mo_2_C. In parallel the peaks at 232.4 eV and 235.4 eV were also observed, which are attributed to (Mo^6+^) Mo3d_5/2_ and Mo3d_3/2_ respectively^46^. This arise due to the surface oxidation of Mo_2_C when exposed to air for prolonged duration^47, 48^. Figure 5c shows high resolution spectrum of C1s. The peak at 284.1 eV is attributable to Mo-C and the peak at 285.4 is assigned to C-C, respectively. In addition O1s spectra in Fig. 5d shows double peaks at 530.2 eV and 531.1 eV correspondin to Mo-O and C-O, respectively^45^.

The full survey XPS spectrum of sample R-6 is shown in Fig. S3.2a (SI). It indicated the presence of Mo3d, C1s, Mo3p and O1s at positions 232.5, 286.7, 397.2, 530.6 eV, respectively. The high resolution spectrum of Mo3d in Fig. S3.2b (SI) revealed that the double peaks at 228.1 eV and 231.3 eV assigned to (Mo^2+^) Mo 3d_5/2_ and Mo3d_3/2_ stemming from Mo_2_C. The double peaks at 229.3 eV shown in insert of (Fig. S3.2b, SI) and 232.7 eV are attributed to (Mo^4+^) Mo3d_5/2_ and Mo3d_3/2_, respectively. The peaks corresponding to 232.4 eV and 235.5 eV are assigned to (Mo^6+^) Mo3d_5/2_ and Mo3d_3/2_, respectively. This is the consequence of surface oxidation of Mo_2_C when exposed to air^48^. The surface oxidation during XPS measurements results in the formation of MoO_x_ mainly composed of MoO_2_ and MoO_3_. The high resolution of C1s spectrum as shown in Fig. S3.2c (SI) reveals two peaks at 284.0 eV and 284.7 eV assigned to Mo-C and C-C respectively. Moreover, the O1s spectrum in Fig. S3.2d (SI) shows presence of three peaks after deconvolution. The peak at 530.1 eV arise due to Mo-O and the peaks at 531.4 eV and 533.9 eV are stemming because of C-O and C=O, respectively. The carbon coated Mo_2_C (R-3 sample) exhibited two oxidation states of Mo corresponding to Mo^2+^ and Mo^6+^. Additional peaks of Mo^4+^ were observed in R-6 sample, which correspond to MoO_2_. The presence of MoO_2_ in R-6 XPS spectrum of Mo3d is in accordance with the results obtained in XRD (Fig. 1b), which also show the presence of MoO_2_ phase in sample.

### Nitrogen adsorption/desorption analysis (BET)

The nitrogen sorption studies were performed to measure the Brunauer-Emmet-Teller (BET) specific surface area of R-3 and R-6 samples. The measured BET specific surface area of carbon coated Mo_2_C (R-3 sample) is 15.7 m^2^g^−1^ along with the mean pore size 33.04 nm and pore volume 0.13 cm^3^g^−1^, respectively. However, R-6 sample exhibits BET specific surface area 7.8 m^2^g^−1^ along with the mean pore size of 39.33 nm and pore volume of 0.07 cm^3^g^−1^, respectively. The data showed that the surface area decreased with the increase in time at particular temperature corresponding to R-3 and R-6 samples, respectively. Moreover, the particle size is increases with increase in the reaction time, which is in agreement with the crystallite size trend calculated with Scherrer equation in XRD pattern (Table 1), which apparently decreases the specific surface area^40, 49^. Figure S3.3 (SI) shows the adsorption isotherms of R3 and R6 samples. As compared to R-3 sample, the inclusion of oxide particle within the synthesized powder R-6 sample decreases the BET surface area and the pore volume, whereas, the mean pore size increases^50^. The adsorption isotherms of both the samples demonstrate the characteristic of a type-II isotherm, as per IUPAC classification^51, 52^. The isotherms observed in the present study shows characteristic of H-4 hysteresis^52, 53^. This indicates that there is formation of complex structure with mesopores and micropores, for both the samples. This phenomenon might be associated with the agglomeration of particles in the final product.

### Formation mechanism of Mo_2_C

The formation of Mo_2_C by reduction-carburization in an autoclave is multistep reduction and carburization process. Based on XRD pattern of all the synthesized samples followed by the microstructural analysis, the formation mechanism of Mo_2_C has been predicted. Mg being highly reactive substance absorbs oxygen from the autoclave while as polypropylene decomposes into the gaseous hydrocarbons in an autoclave^33^. The generation of carburizing atmosphere inside autoclave is rather complex due to the interaction of various gases. Moreover, the exact nature of gases evolved during decomposition of polypropylene in closed autoclave cannot be determined. The MgO being highly reactive catalyst inside the autoclave reduces the gaseous compounds into carbon and hydrogen^49^. The carbon along with hydrogen inside the autoclave is in excess amount. It helps in reduction as well as carburization. The lower reaction temperature of 600 °C for 10 h reaction time reveals that only some reduction of MoO_3_ to intermediate Mo_4_O_11_ specie has taken place. The initial reduction of MoO_3_ to Mo_4_O_11_ in presence of Mg in carbon and hydrogen atmosphere follows a number of reaction paths, giving rise to different reaction products. The possible reactions are given as in appendix S4 (SI) where the most feasible reaction path is:

2 $$4{{\rm{MoO}}}_{3}+{\rm{Mg}}\to {{\rm{Mo}}}_{4}{{\rm{O}}}_{11}+{\rm{MgO}}$$

**Figure 6:**
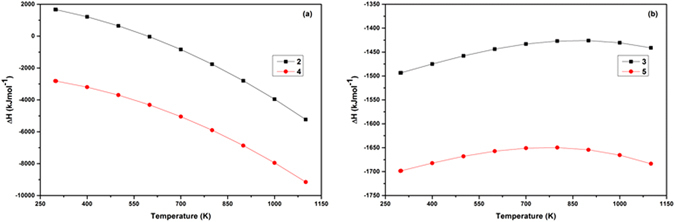
Variation of ∆H with temperature for best possible reactions for (**a**) reduction (**b**) carburization.

The heat of formation (∆H) of the above reactions can be calculated using relations (S4.1 and S4.2, SI)^54, 55^. Variation of ∆H with temperature is shown in Fig. S4 (SI), the reduction of MoO_3_ to Mo_4_O_11_ is most feasible with Mg as reducing agent (2), having more negative ∆H variation with temperature as shown in Fig. 6a. The reaction has less feasibility with higher hydrocarbons as they readily decompose to H and C^34^.

The carbon encapsulates the oxide particles and the hydrogen being small in size penetrates along the grain boundaries and breaks the oxide particles into small constituents which facilitate the reaction in forward direction at faster rate. With the increase in temperature, the formation of Mo_2_C seems to be feasible, the presence of Mo, MoO_2_ and Mo_2_C at temperature of 700 °C for 10 h reveals that the reduction- carburization proceeds in forward direction with increase in temperature. The reduction-carburization of MoO_3_ to Mo_2_C is multistep reduction process as given in appendix S1, (SI) ^31^. The reactions given in appendix C (SI) show some feasible reaction paths for formation of Mo_2_C, Mo, MoO_2_ following the results obtained at 600 °C in hydrogen and carbon atmosphere. The reaction corresponding to reduction- carburization with C and H_2_ (3) is most feasible and the reaction path is given as:

3 $${{\rm{MoO}}}_{3}+{{\rm{Mo}}}_{4}{{\rm{O}}}_{11}+9{\rm{C}}+6{\rm{H}}2\to 2{{\rm{Mo}}}_{2}{\rm{C}}+1/2{{\rm{MoO}}}_{2}+1/2{\rm{Mo}}+3{\rm{CO}}+4{{\rm{H}}}_{2}{\rm{O}}+3{{\rm{CO}}}_{2}+{{\rm{CH}}}_{4}$$

**Figure 7:**
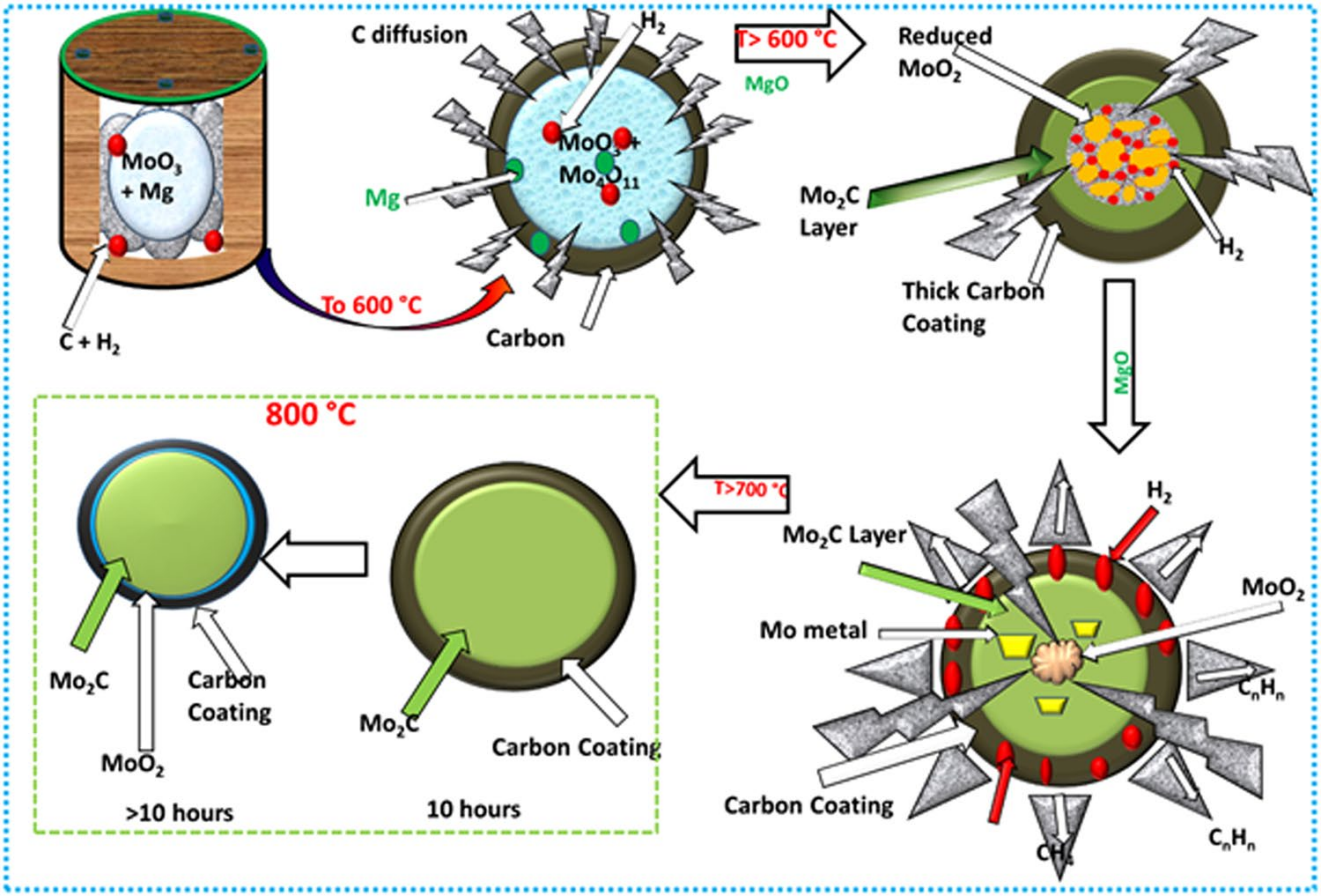
Schematic representation of transformation of MoO_3_ to Mo_2_C nano particles.

The ∆H variation with temperature of above reaction is shown in (Fig. 6b) having negative ∆H at different temperatures and has shown a decreasing trend at higher temperatures predicting more feasibility of phase formation. The ∆H versus temperature graph of the corresponding reactions (appendix S5, SI) having negative ∆H values for all the reactions as shown in Fig. S5a–c (SI), confirms the feasibility of all reaction paths. Certain reaction paths differ only in the byproducts of the reaction, which alter the ∆H value of reaction. Carbon being present in high concentration creates a thick coating around the produced Mo_2_C phase as shown in schematic diagram Fig.7. High carbon concentration also facilitates the direct carburization of MoO_2_ to Mo_2_C with small amount of Mo metal present, which may be because of the enclosed Mo_2_C layer in thick carbon coating that hinders further diffusion of carbon to thoroughly carburize the reduced oxide phase and Mo metal to form single phase Mo_2_C. Some authors suggested the low concentration of CO leads to the formation of metallic Mo, whereas, a high concentration of CO forms Mo_2_C directly from MoO_2_^31, 56^. So the presence of sufficient carbon atoms on the surface needs to travel longer distance to carburize the reduced oxides. Carbon deposit on carbides is higher at lower temperatures. Carburization process may involve other catalytic reactions as CO_2_/H_2_O, reforming of CH_4_ to H_2_ and CO and direct cracking of CH_4_ to H_2_. The decomposition of polypropylene enriches the C_n_H_n_ gases, which react with CO_2_ and H_2_O to reduce their concentration and produce more amount of H_2_, C and CO to enhance the reduction carburization reaction. The enrichment of CO and H_2_ proceed via different path ways^57^, some reaction paths are given in appendix S5.A (SI). The negative ∆G values of the reactions shows the spontaneity of reaction and the lower negative designates the most probable reaction form. Hydrogen produced in reaction also facilitates the carburization reaction by removing excess carbon deposition on the surface by forming hydrocarbon gases. The deposition, removal of carbon (by hydrogen) and carbon dissolution occur simultaneously during reaction process. In order to facilitate the carburization the increase in reaction temperature to 800 °C for 10 h thoroughly reduce the MoO_3_ and Mo_4_O_11_ to MoO_2_ and simultaneously carburize MoO_2_ to Mo_2_C and a single phase Mo_2_C is obtained with excess carbon residue present. The reduction of MoO_3_ and Mo_4_O_11_ to MoO_2_ results via different reaction paths inside an autoclave in H and C atmosphere as given in appendix S6 (SI) and the corresponding graph of ∆H with respect to temperature is given in Fig. S6 (SI). The reaction (4) shows the feasible reaction path having Mg and H_2_ as reducing agents as given below:

4 $${{\rm{MoO}}}_{3}+{{\rm{Mo}}}_{4}{{\rm{O}}}_{11}+{\rm{Mg}}+3{{\rm{H}}}_{2}\to 5{{\rm{MoO}}}_{2}+3{{\rm{H}}}_{2}{\rm{O}}+{\rm{MgO}}$$

The ∆H versus T variation of above reaction is shown in Fig. 6a, having more negative ∆H variation with temperature. All the reactions proceed in presence of Mg as catalyst and favor the reduction of MoO_3_ and MoO_2_ at temperatures greater than 600 °C^58^ which simultaneously get carburized to Mo_2_C. The reaction temperature 800 °C and 10 h time seems to be sufficient to reduce and carburize the MoO_3_ to single phase Mo_2_C. Moreover, different reduction-carburization ﻿reactions of MoO_3_ and Mo_4_O_11_ to Mo_2_C inside an autoclave has been proposed for formation of Mo_2_C in CO, H and C_n_H_n_ atmosphere appendix S7 (SI). The corresponding ∆H versus temperature variation is shown in Fig. S7, (SI) predicting all reactions as feasible pathways because of negative ∆H values at different temperatures up to 800 °C (1073 K). Carburization reaction (5) as a result of C and H_2_ shows the more feasible reaction path given as:

5 $${{\rm{MoO}}}_{3}+{{\rm{Mo}}}_{4}{{\rm{O}}}_{11}+9{{\rm{H}}}_{2}+10{\rm{C}}\to 5/2{{\rm{Mo}}}_{2}{\rm{C}}+6{\rm{CO}}+1/2{{\rm{CH}}}_{4}+8{{\rm{H}}}_{2}{\rm{O}}+{\rm{C}}$$

The ∆H variation with temperature of above reaction as given in (Fig. 6b) shows the feasibility of reaction having low negative ∆H variation with temperature. The formation of Mo_2_C considering the above reaction paths based on the thermodynamic parameters has been schematically represented in Fig. 7. Formation of larger amount of CO_2_ and H_2_O enhances the decarburization reaction. The reaction of surface carbon with CO inside an autoclave results in higher concentration of CO_2_, which favors the diffusion of oxygen in Mo_2_C at higher temperatures as has been schematically represented in Fig. 7. So, increasing the time upto 12 h shows the reversibility of reaction due to presence of large amount of the decarburizing agents CO_2_ and H_2_O as reaction by products.

### Summary

Single phase Mo_2_C nanopowders has been successfully synthesized at 800 °C by single step reduction carburization route in an autoclave in presence of Mg using polypropylene as carbon source. The reaction time of 10 h facilitates the reduction and carburization at different temperatures. The synthesized sample shows good thermal stability in air atmosphere. TGA/DTA results revealed that thermal decomposition of the R-3 sample occurred in six different stages. A multi-stage kinetic analysis was performed to evaluate the activation energy and reaction mechanism by employing Coats-Redfern method. It is observed that the random nucleation (F1 mechanism) dominates the complete thermal decomposition process involved in the R-3 sample. However, in stage 4, the reaction mechanism changes from F1 to F3 mechanism. This may be due to the instantaneous combustion of carbon and oxidation of molybdenum oxide intermediates. The average particle size of sample R-3 observed from TEM micrographs (40–50 nm) is more close to that obtained from the UDM model, which shows better fit than other two models of W-H analysis. Particles are encapsulated within carbon matrix and are highly agglomerated. The HRTEM micrograph clearly identifies the presence of graphitic carbon over Mo_2_C. The interaction of various gases inside autoclave produces H_2_, C_n_H_n_ and CO that favors reduction-carburization of MoO_3_ and provides the carbonaceous atmosphere to reaction process. CO_2_ and H_2_O are also produced as reaction byproducts. The thermodynamic study indicates that carburization is most favorable at low temperature in presence of Mg. The utilization of polypropylene as carbon source makes the process cost effective and provides the path to utilize plastic wastes as carbon source for preparation of useful materials. The process will be of high industrial use and an alternate to recycling of plastic wastes to overcome the environmental issues.

### Experimental Details

#### Synthesis

For the synthesis of nano crystalline Mo_2_C, the mixture of MoO_3_ (1.4394 g), Mg (3.5 g) and polypropylene (1 g) was mixed with the help of agate-mortar. Polypropylene has not been used so far for the synthesis of carbides. The above mixture was put in a specially designed autoclave^59^. The autoclave was sealed properly and put in a furnace at room temperature. The temperature of the furnace was raised from room temperature to desired temperature 600 °C, 700 °C and 800 °C at constant heating rate of 5 °C per minute and maintained at that temperature for 2, 5, 10 and 12 hours. After that autoclave was allowed to cool to room temperature and the obtained product was collected and washed with (1:1) diluted HCl to remove the MgO. The resultant powder was washed many times to remove the HCl followed by vacuum drying at 120 °C.

#### Characterization

The as prepared samples were characterized by XRD using PANalytical X-Pert-Pro with CuKα radiation (λ = 1.5406 Å) attained from copper target using an inbuilt Ni filter. The X-ray powder diffraction data of all samples was collected at room temperature between 20° ≤ 2θ ≤ 80° with a step size of 0.0130° (2θ). The ICDD data base (using X-Pert High Score Plus) was used for phase identification and further confirmation was done with the help of Rietveld refinement of the pure phase XRD patterns (using Full Prof Suite). TG/DTA and DTG (*Exstar TG/DTA 6300*) analysis was done in air atmosphere from room temperature to 1000 °C at a heating rate of 5 °C/min. Kinetic analysis has been done to study the thermal decomposition of the prepared sample and kinetic theory is presented in appendix G (SI). Coats-Redfern method has been applied to determine the decomposition mechanism of the product phase^28^. The activation energy (*E*_a_) and pre-exponential factor (*A*) has also been calculated. The morphological and microstructural features of synthesized Mo_2_C powders was analyzed with field- emission scanning electron microscope (FE-SEM) (*Hitachi SU 8010*) and transmission electron microscope (*TEM) (JEOL 2100* *F*) operating at 15 kV and 200 kV respectively. To elucidate the surface chemical compositions and their valence states, the carbon coated Mo_2_C samples were analyzed by X-ray photoelectron spectroscopy (XPS). The measurements were carried out using XPS spectrometer (ESCA+) using Al-Kα radiation (1486.7 eV), operating at 15 kV and 15 mA. The C1s peak 284.6 eV was selected as reference to calibrate the position of other peaks. The nitrogen sorption studies for surface analysis were conducted at 77 K to determine Brunauer-Emmet-Teller (BET) surface area, the pore size and the pore volume using BEL-miniII.

## Electronic supplementary material

Supplementary Information
