# Supplementary material for: Thermal and structural studies of carbon coated Mo2C synthesized via in-situ single step reduction-carburization
Source: Sci Rep. 2017 Jun 14;7:3518. doi: 10.1038/s41598-017-03197-8 (PMC5471264; doi:10.1038/s41598-017-03197-8)
Supplement: Supplementary file 1 — Supplementary Information [file 41598_2017_3197_MOESM1_ESM.pdf]

Supplementary Information (SI)

Thermal and structural studies of carbon coated Mo_2C synthesized via in-situ single step reduction-carburization

Rameez Ahmad Mir, Piyush Sharma and Om Prakash Pandey*

School of Physics and Materials Science, Thapar University, Patiala- 147 004, India

E-mail: *oppandey@thapar.edu

Appendix S 1

$$MoO_3 \rightarrow MoO_2 \rightarrow Mo + Mo_2C \quad \textbf{(S 1.1)}$$

$$MoO_3 \rightarrow MoO_2 \rightarrow Mo_2C \quad \textbf{(S 1.2)}$$

Firstly, reduction of MoO_3 to MoO_2 followed by MoO_2 to Mo_2C. The reduction of MoO_2 to Mo_2C can occur through an intermediate product of metallic Mo as shown in equation S1.1. Sometimes reaction proceeds without formation of metallic phase depending on reaction conditions as given in equation S1.2 and is more favorable for endothermic solid state reactions.

Scherrer formula

The crystallite size obtained from Scherrer equation is volume weighted quantity and uses full width at half maxima for determination of crystallite size, D [38]:

$$D = \frac{k\lambda}{\beta Cos\theta} \Rightarrow Cos\,\theta = \frac{k\lambda}{D}\left(\frac{1}{\beta}\right) \quad \textbf{(S 1.3)}$$

Where, k is shape factor, λ is wavelength of X-ray radiation, D is effective crystallite size in nanometers, β is peak width corrected for instrumental broadening as described in equation (1) and θ is Bragg angle.

Williamson Hall analysis

Stokes and Wilson assigned a mathematical relation for peak broadening solely due to strain [1]. Crystal imperfection and distortion constitute the strain broadening and the induced strain is given as follows:

$$\boldsymbol{\beta = 4\,\varepsilon\,tan} \quad \textbf{(S 1.4)}$$

As Scherrer equation follows 1/cosθ dependency, the Williamson-Hall method varies with tanθ and allows the separation of diffracted beams when both crystallite size and strain are simultaneously involved [38]. Even though β is combination of equation (S1.3) and (S1.4), Williamson Hall plot is used to separate the individual effects:

$$\boldsymbol{\beta Cos\theta} = \left[\frac{\boldsymbol{k\lambda}}{\boldsymbol{D}}\right] + [\boldsymbol{4\varepsilon\, Sin\theta}] \quad \textbf{(S 1.5)}$$

Equation (S1.5) represents the uniform deformation model where strain was considered to be isotropic through whole crystal.

In many cases the strain homogeneity assumption and isotropy is not fulfilled, so uniform stress deformation model and uniform deformation energy density model come into consideration.

The anisotropic nature of Young's modulus is considered by both the models, hence are more realistic routes [2, 39]. In uniform deformation stress model, anisotropic micro strain ε is exhibited due to isotropic stress σ. The linear proportionality between stress and strain is given by Hook's law, $\sigma = \varepsilon E_{hkl}$. Considering this, Williamson-Hall equation has been formulated as:

$$\boldsymbol{\beta} = \left(\frac{\boldsymbol{k\lambda}}{\boldsymbol{D}}\right) + \left(\frac{\boldsymbol{4\sigma\, Sin\theta}}{\boldsymbol{E_{hkl}}}\right) \quad \textbf{(S 1.6)}$$

Where E_{hkl} is Young modulus and for hexagonal crystal phases, it can be calculated by following relation: [5]

$$\boldsymbol{E_{hkl}} = \left(\boldsymbol{h^2} + \frac{(\boldsymbol{h+2k})^2}{3} + \left(\frac{\boldsymbol{al}}{\boldsymbol{c}}\right)^2\right)^2 \times \left[\boldsymbol{S_{11}}\left(\boldsymbol{h^2} + \frac{(\boldsymbol{h+2k})^2}{3}\right)^2 + \boldsymbol{S_{33}}\left(\frac{\boldsymbol{al}}{\boldsymbol{c}}\right)^4 + (\boldsymbol{2S_{13}} + \boldsymbol{S_{44}})\left(\left(\boldsymbol{h^2} + \frac{(\boldsymbol{h+2k})^2}{3}\right)\left(\frac{\boldsymbol{al}}{\boldsymbol{c}}\right)^2\right)\right]^{-1} \quad \textbf{(S 1.7)}$$

Where S_{11}, S_{13}, S_{33} and S_{44} are elastic compliances of Mo_2C having values of 2.4×10^{-3}, -0.68×10^{-3}, 2.56×10^{-3} and 7.353×10^{-3} GPa^{-1} respectively [3-4]. Young modulus was calculated as 390.49 GPa which is in close approximation to earlier reported by [5]. Plotting (4 Sinθ /E_{hkl}) on x-axis and (β Cosθ) on y axis as shown in Fig. S2.a, the value of stress was calculated from slope of fitted line and crystallite size is extracted from intercept reported in Table A2. The uniform deformation energy density model assumes isotropic energy density as a cause of anisotropic deformation within the crystal. The energy density u as a function of strain for a system that follows Hook's law is given by $u = \varepsilon^2 E_{hkl}/2$. According to energy and strain relation, the uniform deformation energy model is given by following mathematical relation:

$$\boldsymbol{\beta = \left(\frac{k\lambda}{D}\right) + \left(4\ Sin\theta \left(\frac{2u}{E_{hkl}}\right)^{\frac{1}{2}}\right)} \qquad \textbf{(S 1.8)}$$

Plots were drawn with 4 Sinθ $(2u/E_{hkl})^{1/2}$ and β Cosθ on x-axis and y-axis respectively and data fitted to lines as shown in (Fig. S2.b). The slope of the fitted line is used to estimate the value of energy density u while as crystallite size D is estimated from intercept as given in Table A2. The deformation stress and deformation energy density are related as $u = \sigma^2/E_{hkl}$.

At constant temperature, with increase in synthesis time R-4 (2h), R-5 (5h), R-3 (10h) and R-6 (12h), the strain value increases upto 10h while, the value decreases when synthesis time is increased up to 12h where the carbon drain out from the system and the product is converted back to MoO_2, showing the reversibility of the reaction.

Table S 1. Williamson Hall analysis Mo_2C phase obtained samples**.**

Sample ID	USDM			USEDM			
	D (nm)	σ (GPa)	$\varepsilon \times 10^{-4}$	D (nm)	σ (GPa)	$\varepsilon \times 10^{-4}$	$U \times 10^2$ (KJm^{-3})
R-2	54.37	0.2654	6.82	56.36	0.2876	7.38	1.06
R-3	45.76	0.2059	5.29	46.68	0.2182	5.60	0.61
R-4	37.88	0.1563	4.01	38.09	0.1612	4.13	0.33
R-5	51.74	0.1433	3.68	51.74	0.1580	4.05	0.32
R-6	96.28	0.1662	6.00	96.287	0.1676	4.30	0.36

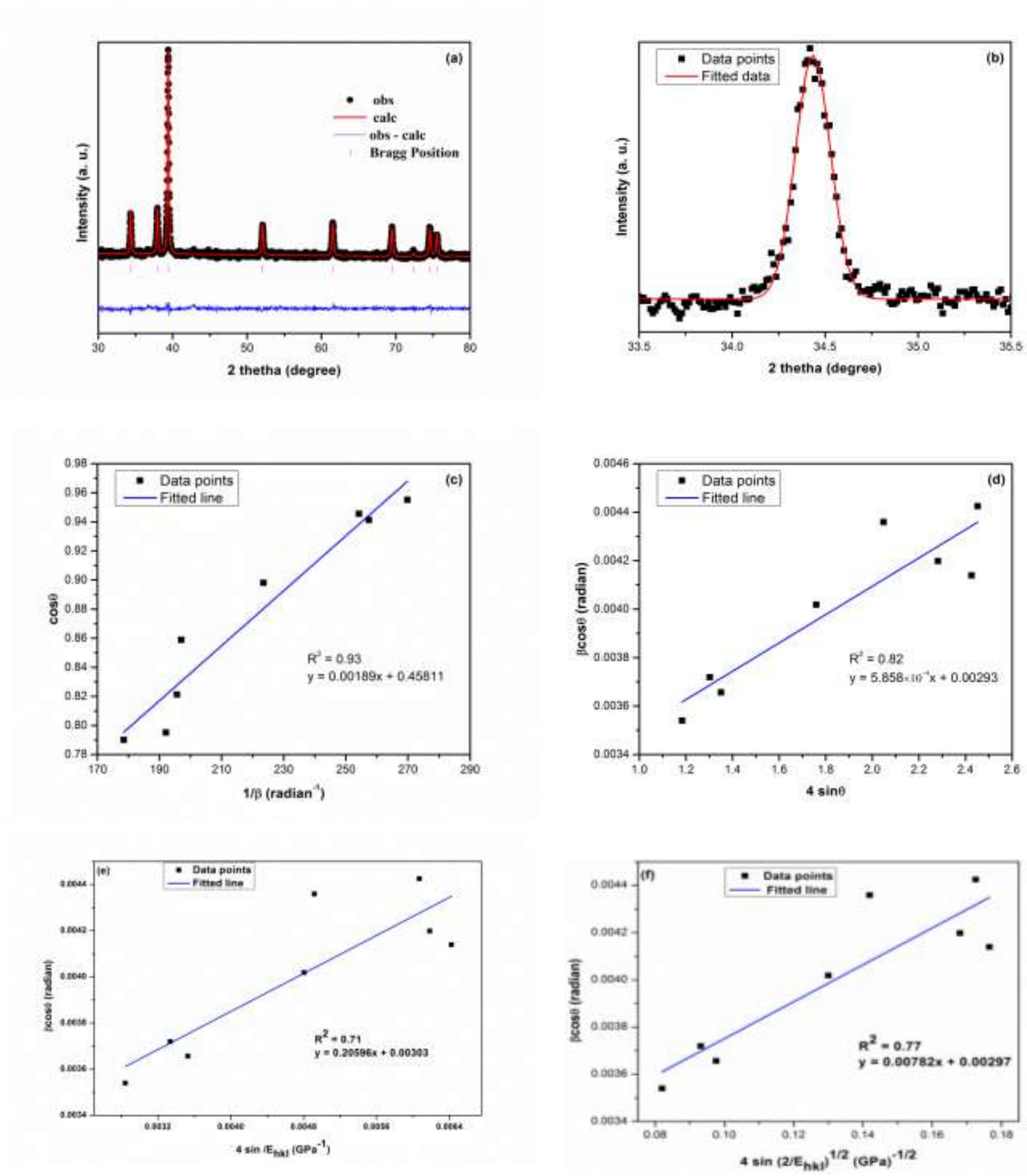

Fig. S 1 Rietveld refinement plot and W-H analysis of (R-3 sample). (a) The measured and calculated data are observed by dots and solid curve respectively. The plot below shows the difference between measured and calculated data, whereasas the tick marks indicate the bragg positions. (b) Gaussian fitted data, (c) Sherrer plot of sample, (d) UDM, (e) USDM and (f) USEDM model. , the crystalline size is extracted from y-intercept of fit, strain and deformation energy density id is estimated from slope of fit.

Appendix S 2

Table S 2. Various reaction mechanisms involved in a thermal decomposition process.

**Reaction Mechanism**	**Integral form** $\boldsymbol{g(\alpha)}$
**Diffusion mechanism**	
D_1 (1 – D diffusion)	α^2
D_2 (2 – D diffusion)	$\alpha + (1-\alpha)\ (ln(1-\alpha))$
D_3 (3 – D diffusion)	$(1-(1-\alpha)^{1/3})^2$
D_4 (Ginstling-Brounshtein)	$1-2\alpha/3-(1-\alpha)^{2/3}$
**Random nucleation mechanism**	
F_1 (First order)	$-ln(1-\alpha)$
F_2 (Second order)	$1/(1-\alpha)$
F_3 (Third order)	$1/2(1-\alpha)$
**Nucleation and growth mechanism**	
A_2 (Avrami-Erofeyev)	$(-ln(1-\alpha))^{1/2}$
A_3 (Avrami-Erofeyev)	$(-ln(1-\alpha))^{1/3}$
A_4 (Avrami-Erofeyev)	$(-ln(1-\alpha))^{1/4}$
**Phase boundary controlled reaction mechanism**	
R_1 (1-D movement)	α
R_2 (contracting area)	$1-(1-\alpha)^{1/2}$
R_3 (contracting volume)	$1-(1-\alpha)^{1/3}$

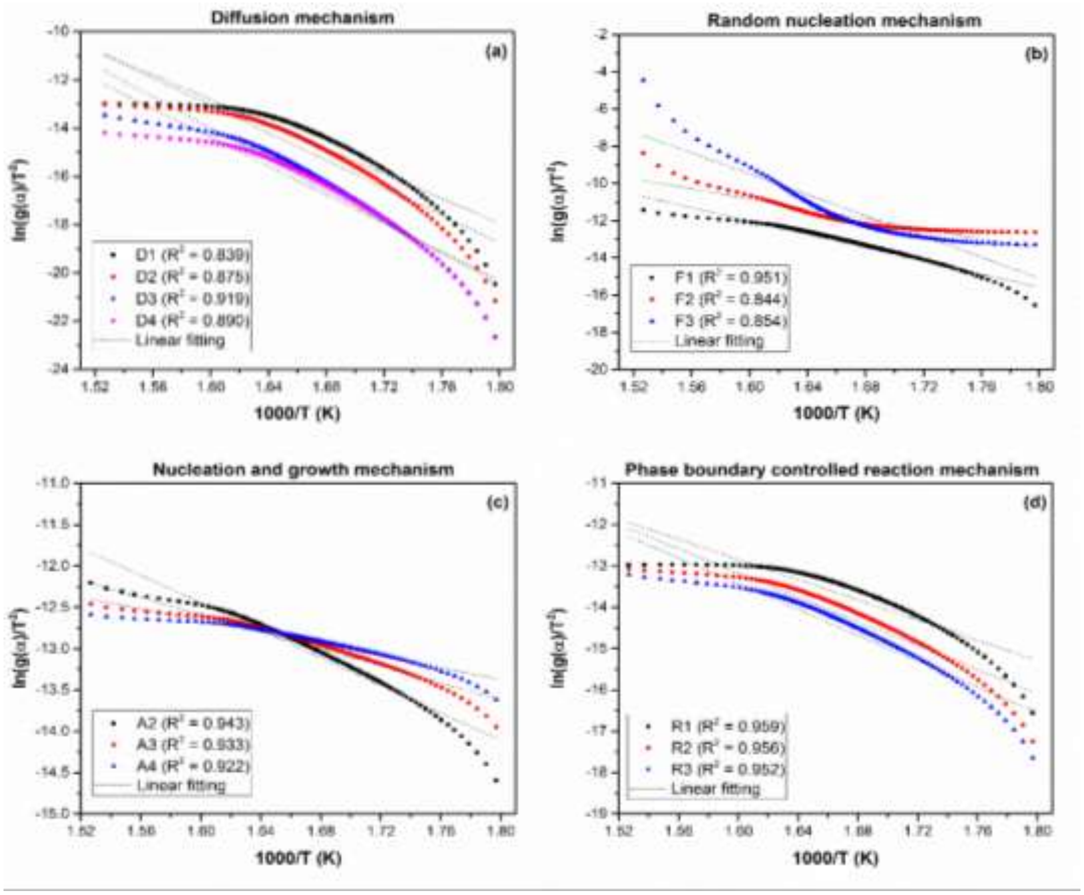

Fig. S 2.1 Linear fitted curves of stage 2 corresponding to (a) Diffusion mechanism (b) Random nucleation mechanism (c) Nucleation and growth mechanism and (d) Phase boundary controlled reaction mechanism.

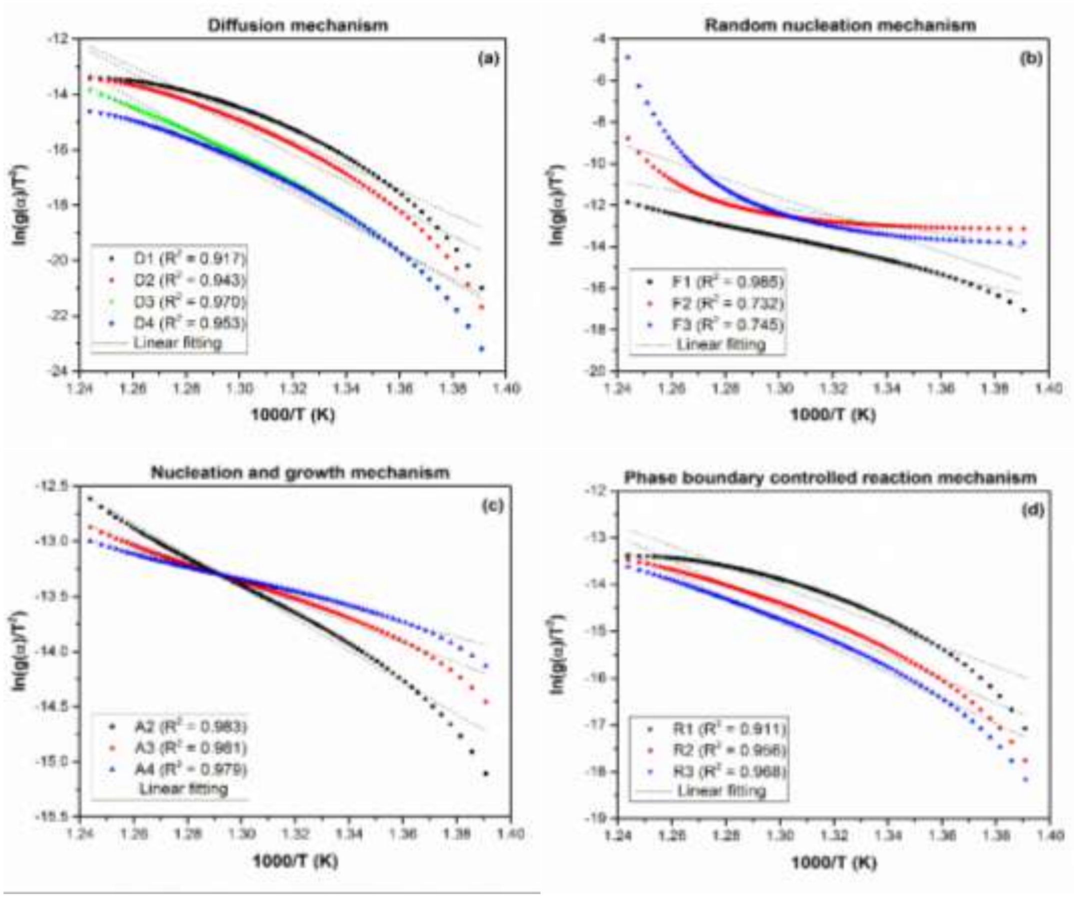

Fig. S 2.2 Linear fitted curves of stage 3 corresponding to (a) Diffusion mechanism (b) Random nucleation mechanism (c) Nucleation and growth mechanism and (d) Phase boundary controlled reaction mechanism.

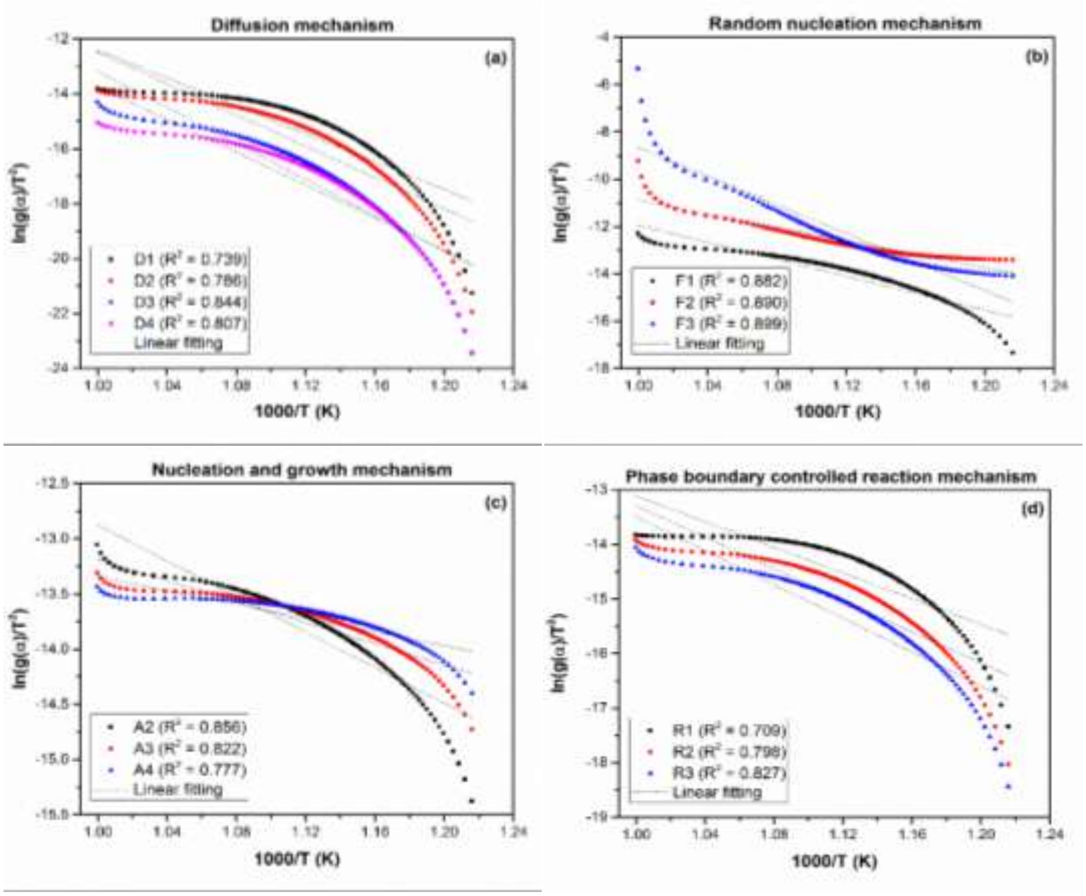

Fig. S 2.3 Linear fitted curves of stage 4 corresponding to (a) Diffusion mechanism (b) Random nucleation mechanism (c) Nucleation and growth mechanism and (d) Phase boundary controlled reaction mechanism.

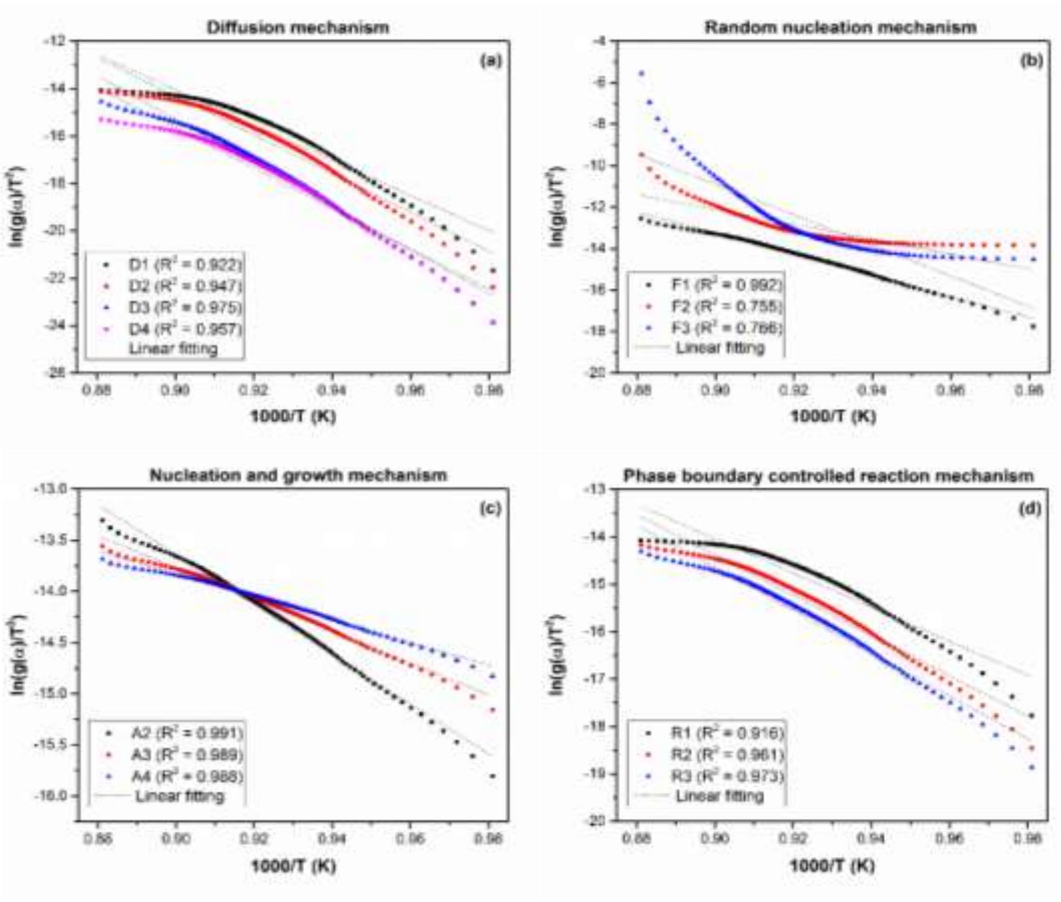

Fig. S 2.4 Linear fitted curves of stage 5 corresponding to (a) Diffusion mechanism (b) Random nucleation mechanism (c) Nucleation and growth mechanism and (d) Phase boundary controlled reaction mechanism.

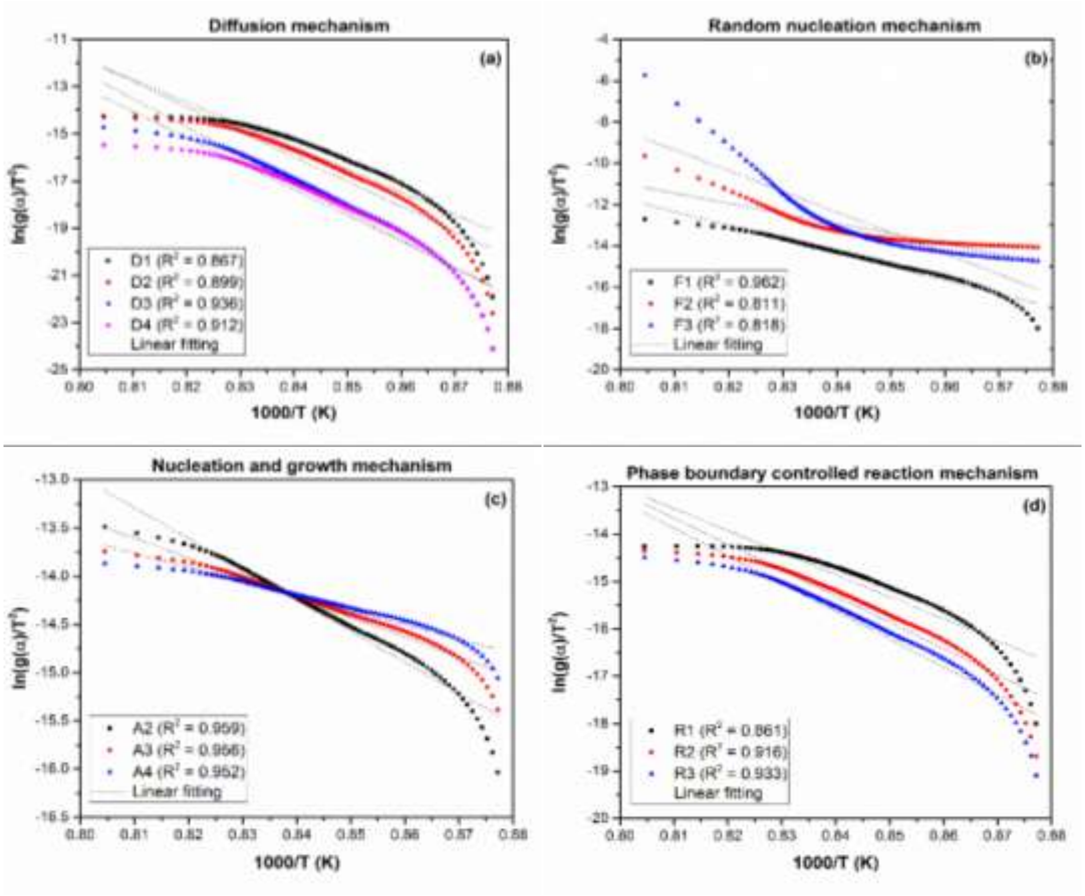

Fig. S 2.5 Linear fitted curves of stage 6 corresponding to (a) Diffusion mechanism (b) Random nucleation mechanism (c) Nucleation and growth mechanism and (d) Phase boundary controlled reaction mechanism.

Appendix S 3

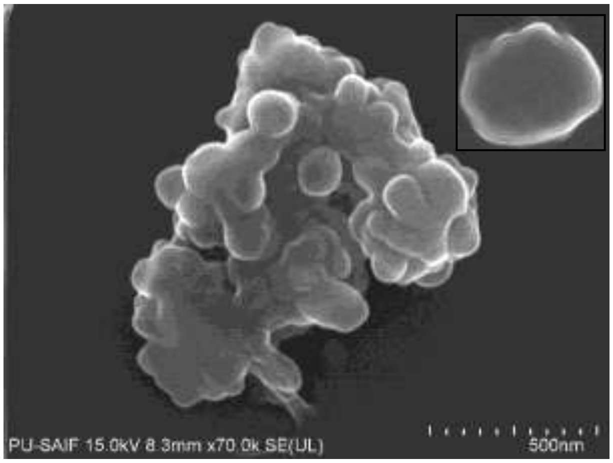

Fig. S 3 FESEM micrograp of Mo_2C nanopowder R-3 sample showing spherical/faceted morphology.

The log-normal probability distribution function is given by following equation:

$$f(d) = \frac{1}{\sqrt{2\pi} d log\sigma_s} exp\left\{-\frac{(\log d_i - log\mu_s)^2}{2(\log \sigma_s)^2}\right\} \quad \textbf{(S 3.1)}$$

Where, $log\sigma_s = \sqrt{\frac{\sum(logd_i - log\mu_s)^2}{\sum n_i}}$ **(S 3.2)**

$$log\mu_s = \frac{\sum d_i}{\sum n_i} \quad \textbf{(S 3.3)}$$

Where, $f(d)$ signifies the log-normal distribution, d_i particle diameter, n_i is the number of particles, μ_s is the mean diameter and σ_s is the standard deviation respectively.

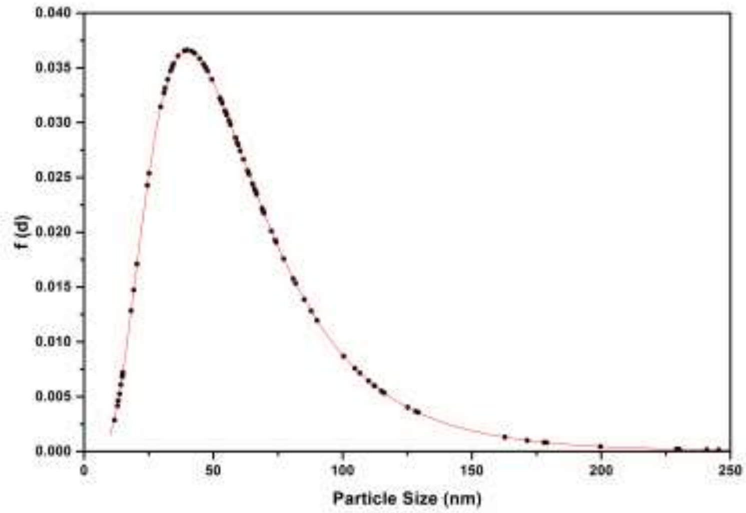

Fig. S 3.1 Particle size distribution from TEM using log-normal probability distribution function.

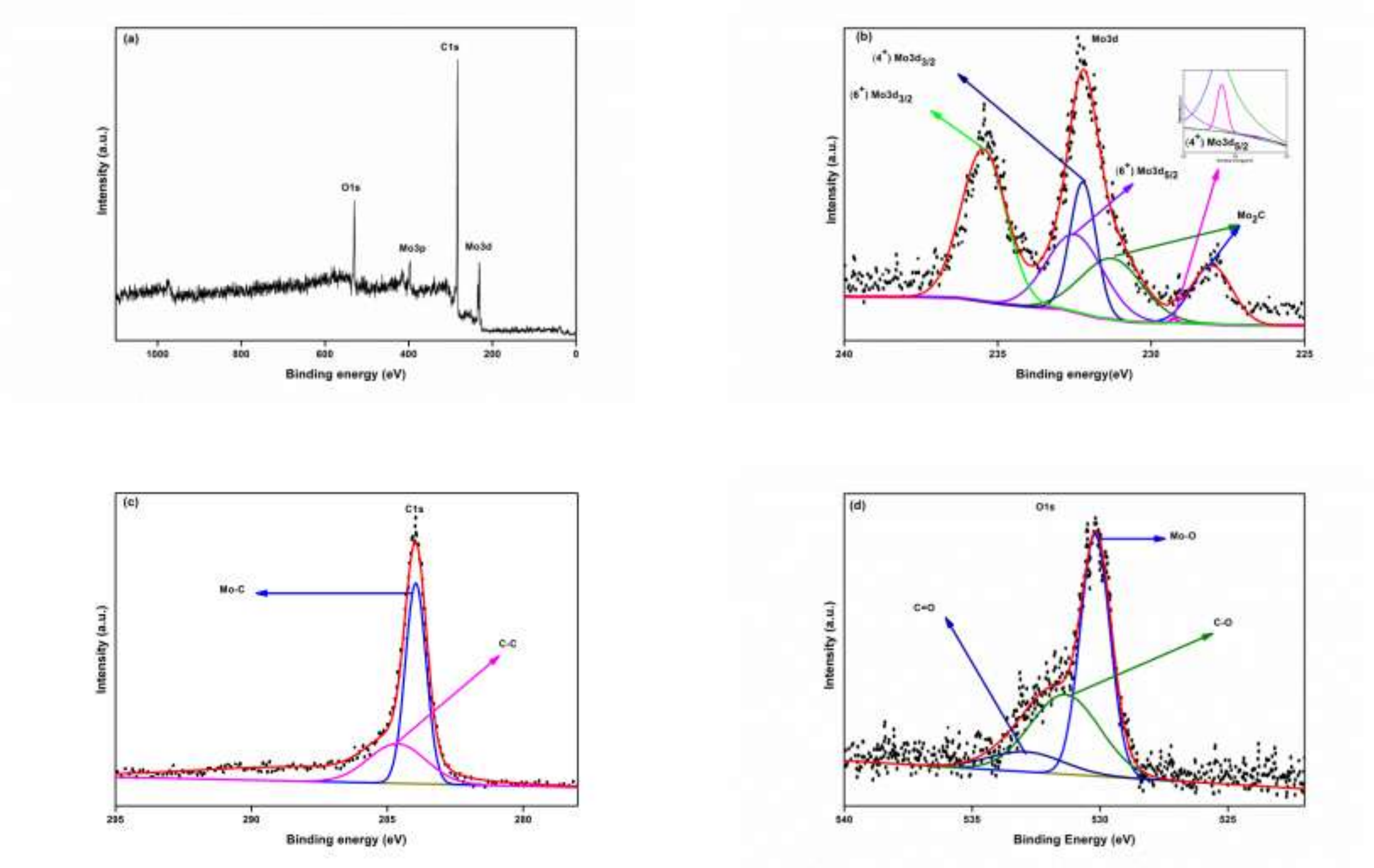

Fig. S 3.2 XPS spectrum of R-6 sample (a) full survey (b) Mo3d (c) C1s and (d) O1s spectrum

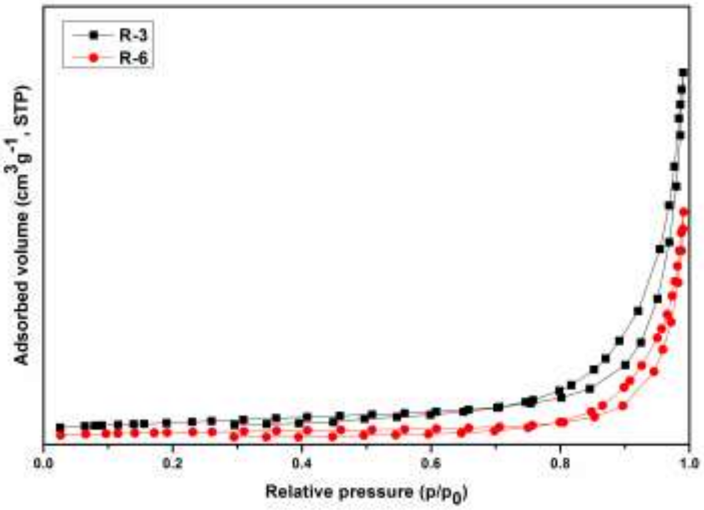

Fig S 3.3 Nitrogen adsorption/desorption isotherm of R-3 and R-6 samples

Appendix S 4

$$\Delta H = \Delta H_{298} + \int_{298}^{T} C_p \, dt \qquad \text{(S 4 .1)}$$

$$C_p = a + b10^{-3}T + C10^{6}T^{-2} + d10^{-6}T^{2} \qquad \text{(S 4.2)}$$

$4MoO_3 + Mg \rightarrow Mo_4O_{11} + MgO$ (S 4.3)

$4MoO_3 + 1/2H_2 + 1/2Mg \rightarrow Mo_4O_{11} + 1/2MgO + 1/2\ H_2O$ (S 4.4)

$4MoO_3 + 1/2C + 1/2Mg \rightarrow Mo_4O_{11} + 1/2MgO + ½CO$ (S 4.5)

$4MoO_3 + 1/2CO + 1/2Mg \rightarrow Mo_4O_{11} + 1/2MgO + 1/2CO_2$ (S 4.6)

$4MoO_3 + 1/16C_2H_6 + 11/16Mg \rightarrow Mo_4O_{11} + 11/16MgO + 1/8CO + 3/16H_2O$ (S 4.7)

$4MoO_3 + 1/8C_2H_4 + 1/2Mg \rightarrow Mo_4O_{11} + 1/2MgO + 1/4CO + 1/4H_2O$ (S 4.8)

$4MoO_3 + 1/4CH_4 + 1/4Mg \rightarrow Mo_4O_{11} + 1/4MgO + 1/4CO + 1/2H_2O$ (S 4.9)

$4MoO_3 + 1/9C_3H_6 + 3/9Mg \rightarrow Mo_4O_{11} + 3/9MgO + 1/3CO + 3/9H_2O$ (S 4.10)

$4MoO_3 + 1/9C_3H_8 + 2/9Mg \rightarrow Mo_4O_{11} + 2/9MgO + 1/3CO + 8/18H_2O$ (S 4.11)

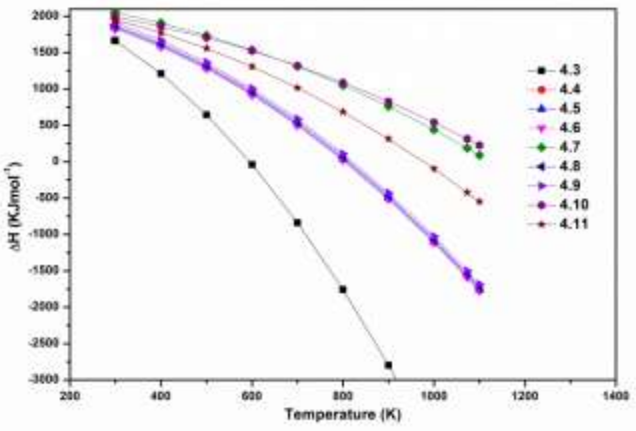

Fig. S 4 Variation in heat of formation with temperature for reduction of MoO_3 to Mo_4O_{11}. It also shows feasibility with both Mg+ H and Mg+ C. However, the reduction of Mg+H (23) and Mg+C (24) is almost similar as the data points overlap. The variation of ΔH with temperature of above reactions is shown in Fig. S4.

Appendix S 5

$Mo_4O_{11} + MoO_3 + 11C \rightarrow 2Mo_2C + 1/2\ MoO_2 + 1/2Mo + 4CO_2 + 5CO$ (S 5.1)

$Mo_4O_{11} + MoO_3 + 6C + 5H_2 \rightarrow 2Mo_2C + 1/2MoO_2 + 1/2Mo + 4CO_2 + 5H_2O$ (S 5.2)

$Mo_4O_{11} + MoO_3 + 8C + 7H_2 \rightarrow 2Mo_2C + 1/2MoO_2 + 1/2Mo + 6CO + 7H_2O$ (S 5.3)

$Mo_4O_{11} + MoO_3 + 8C + 4H_2 \rightarrow 2Mo_2C + 1/2MoO_2 + 1/2Mo + 3CO + 4H_2O + 3CO_2$ (S 5.4)

$Mo_4O_{11} + MoO_3 + 9C + 4H_2 \rightarrow 2Mo_2C + 1/2MoO_2 + 1/2Mo + 4CO + 3H_2O + 3CO_2 + H_2$ (S 5.5)

$Mo_4O_{11} + MoO_3 + 9C + 4H_2 \rightarrow 2Mo_2C + 1/2MoO_2 + 1/2Mo + 3CO + 4H_2O + 3CO_2 + C$ (S 5.6)

$Mo_4O_{11} + MoO_3 + 9C + 6H_2 \rightarrow 2Mo_2C + 1/2MoO_2 + 1/2Mo + 3CO + 4H_2O + 3CO_2 + CH_4$ (S 5.7)

$Mo_4O_{11} + MoO_3 + 8CO + 9H_2 \rightarrow 2Mo_2C + 1/2MoO_2 + 1/2Mo + 9H_2O + 6CO_2$ (S 5.8)

$Mo_4O_{11} + MoO_3 + 6CO + 13H_2 \rightarrow 2Mo_2C + 1/2MoO_2 + 1/2Mo + 2CO + 13H_2O + 2CO_2$ (S 5.9)

$Mo_4O_{11} + MoO_3 + 9CO + 15H_2 \rightarrow 2Mo_2C + 1/2MoO_2 + 1/2Mo + 3CO + 13H_2O + 3CO_2 + CH_4$ (S 5.10)

$MoO_3 + Mo_4O_{11} + 5CH_4 \rightarrow 2Mo_2C + 1/2MoO_2 + 1/2Mo + 7H_2O + 3CO_2 + 3H_2$ (S 5.11)

$MoO_3 + Mo_4O_{11} + 7CH_4 \rightarrow 2Mo_2C + 1/2MoO_2 + 1/2Mo + 5H_2O + 2CO + 3CO_2 + 9H_2$ (S 5.12)

$MoO_3 + Mo_4O_{11} + 8CH_4 \rightarrow 2Mo_2C + 1/2MoO_2 + 1/2Mo + 8H_2O + 5CO + CH_4 + 6H_2$ (S 5.13)

$MoO_3 + Mo_4O_{11} + 4CH_4 + H_2 \rightarrow 2Mo_2C + 1/2MoO_2 + 1/2Mo + 2CO_2 + 9H_2O$ (S 5.14)

$MoO_3 + Mo_4O_{11} + 4CH_4 + 2H_2 \rightarrow 2Mo_2C + 1/2MoO_2 + 1/2Mo + CO_2 + CO + 10H_2O$ (S 5.15)

$MoO_3 + Mo_4O_{11} + 5CH_4 + 2H_2 \rightarrow 2Mo_2C + 1/2MoO_2 + 1/2Mo + CO_2 + CO + 10H_2O + CH_4$ (S 5.16)

$MoO_3 + Mo_4O_{11} + 5C_2H_4 \rightarrow 2Mo_2C + 1/2MoO_2 + 1/2Mo + 2CO_2 + 9H_2O + 6C + H_2$ (S 5.17)

$MoO_3 + Mo_4O_{11} + 4C_2H_4 \rightarrow 2Mo_2C + 1/2MoO_2 + 1/2Mo + 3CO_2 + 4H_2O + 3CO + 4H_2$ (S 5.18)

$MoO_3 + Mo_4O_{11} + 3C_2H_6 \rightarrow 2Mo_2C + 1/2MoO_2 + 1/2Mo + 4CO_2 + 5H_2O + 4H_2$ (S 5.19)

$MoO_3 + Mo_4O_{11} + 3C_2H_6 \rightarrow 2Mo_2C + 1/2MoO_2 + 1/2Mo + 2CO + 2CO_2 + 7H_2O + 2H_2$ (S 5.20)

$MoO_3 + Mo_4O_{11} + 4C_2H_6 \rightarrow 2Mo_2C + 1/2MoO_2 + 1/2Mo + 2CO + 3CO_2 + 5H_2O + CH_4 + 5H_2$ (S 5.21)

$MoO_3 + Mo_4O_{11} + 3C_3H_6 \rightarrow 2Mo_2C + 1/2MoO_2 + 1/2Mo + 4CO + 3CO_2 + 3H_2O + 6H_2$ (S 5.22)

$MoO_3 + Mo_4O_{11} + 3C_3H_6 \rightarrow 2Mo_2C + 1/2MoO_2 + 1/2Mo + 2CO + 3CO_2 + 5H_2O + 2CH_4$ (S 5.23)

$MoO_3 + Mo_4O_{11} + 3C_3H_6 \rightarrow 2Mo_2C + 1/2MoO_2 + 1/2Mo + 2CO + 2CO_2 + 7H_2O + 3C + 2H_2$ (S 5.24)

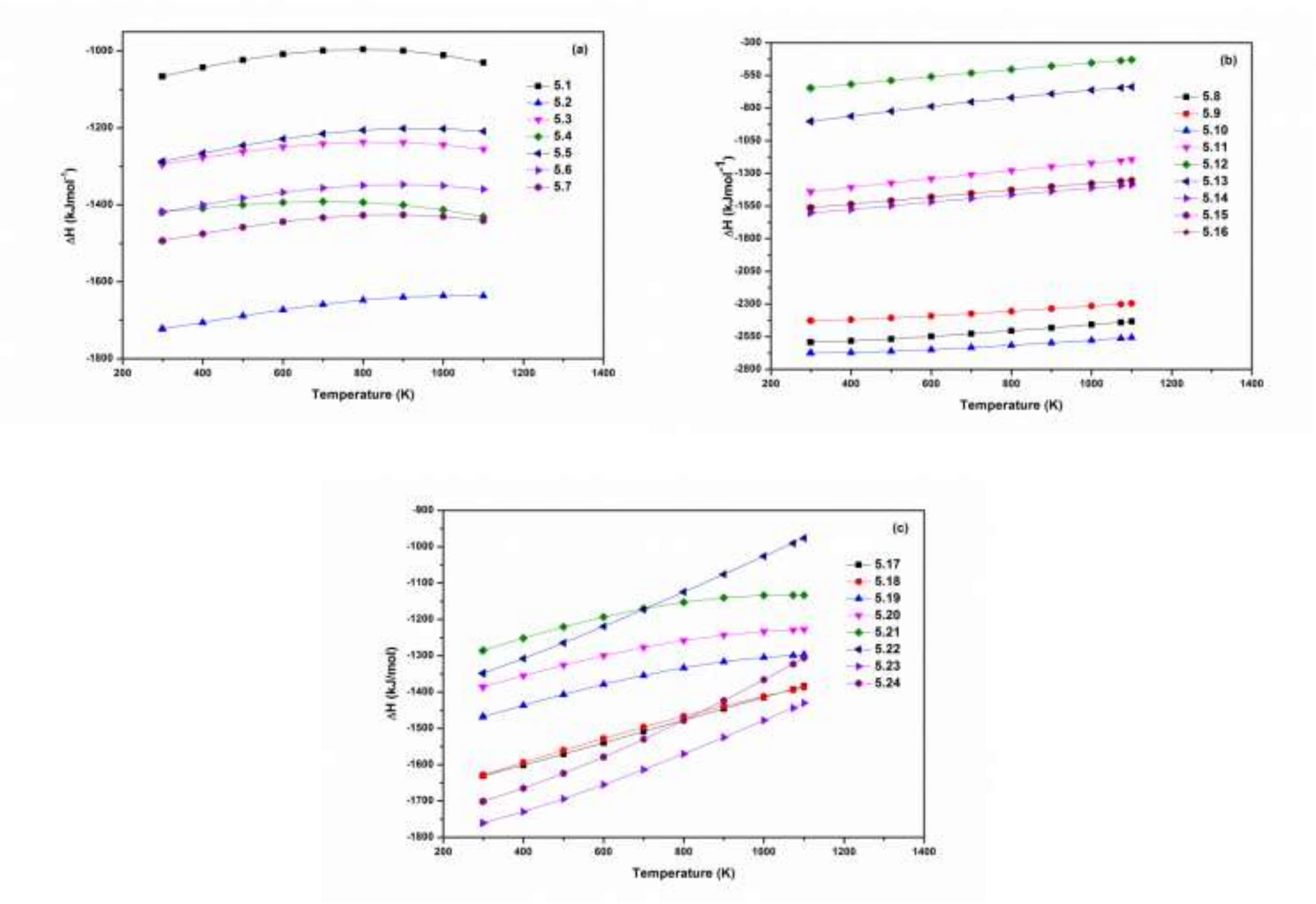

Fig. S 5 Variation ~~with~~ in heat of formation with temperature for reduction-carburization of MoO_3+Mo_4O_{11} to Mo_2C, MoO_2 and Mo.

Appendix S 5.A

$C + CO_2 \rightarrow 2CO$	$\Delta H = +172.5 KJ/mol^{-1}$, $\Delta G = -16.2 KJ/mol^{-1}$	(S 5A.1)
$C + H_2O \rightarrow CO + H_2$	$\Delta H = +131.3 KJ/mol^{-1}$, $\Delta G = -12.3 KJ/mol^{-1}$	(S5A.2)
$C_2H_2 \rightarrow 2C + H_2$	$\Delta H = -226.7 KJ/mol^{-1}$, $\Delta G = -163.7 KJ/mol^{-1}$	(S 5A.3)
$C_2H_6 \rightarrow C_2H_4 + 2H_2$	$\Delta H = +134.94 KJ/mol^{-1}$, $\Delta G = -17.65 KJ/mol^{-1}$	(S 5A.4)
$6CH_4 + O_2 \rightarrow 2C_2H_2 + 2CO + 10H_2$	$\Delta H = +681.3 KJ/mol^{-1}$, $\Delta G = -157.1 KJ/mol^{-1}$	(S 5A.5)
$CH_4 + CO_2 \rightarrow 2CO + 2H_2$	$\Delta H = +260.76 KJ/mol^{-1}$, $\Delta G = -43.08 KJ/mol^{-1}$	(S 5A.6)
$CH_4 + 2O_2 \rightarrow CO_2 + 2H_2O$	$\Delta H = -802 KJmol^{-1}$, $\Delta G = -796.8 KJmol^{-1}$	(S 5A.7)
$CH_4 \rightarrow C + 2H_2$	$\Delta H = -74.8 KJmol^{-1}$, $\Delta G = +11.9 KJmol^{-1}$	(S 5A.8)
$CO + 3H_2 \rightarrow CH_4 + H_2O$	$\Delta H = -250.1 KJmol^{-1}$, $\Delta G = +107.8 KJmol^{-1}$	(S 5A.9

Appendix S 6

$$MoO_3 + Mo_4O_{11} + 4H_2 \rightarrow 5MoO_2 + 4H_2O \tag{S 6.1}$$

$$MoO_3 + Mo_4O_{11} + Mg + 3H_2 \rightarrow 5MoO_2 + 3H_2O + MgO \tag{S 6.2}$$

$$MoO_3 + Mo_4O_{11} + 3CO + 4H_2 \rightarrow 5MoO_2 + 3CO + 4H_2O \tag{S 6.3}$$

$$MoO_3 + Mo_4O_{11} + 2CO + 2H_2 \rightarrow 5MoO_2 + 2CO_2 + 2H_2O \tag{S 6.4}$$

$$MoO_3 + Mo_4O_{11} + CH_4 \rightarrow 5MoO_2 + 2CO_2 + H_2O \tag{S 6.5}$$

$$MoO_3 + Mo_4O_{11} + CH_4 + H_2 \rightarrow 5MoO_2 + CO_2 + 2H_2O + H_2 \tag{S 6.6}$$

$$MoO_3 + Mo_4O_{11} + C_2H_4 \rightarrow 5MoO_2 + 2CO_2 + 2H_2 \tag{S 6.7}$$

$$MoO_3 + Mo_4O_{11} + C_2H_4 \rightarrow 5MoO_2 + 2CO + 2H_2O \tag{S 6.8}$$

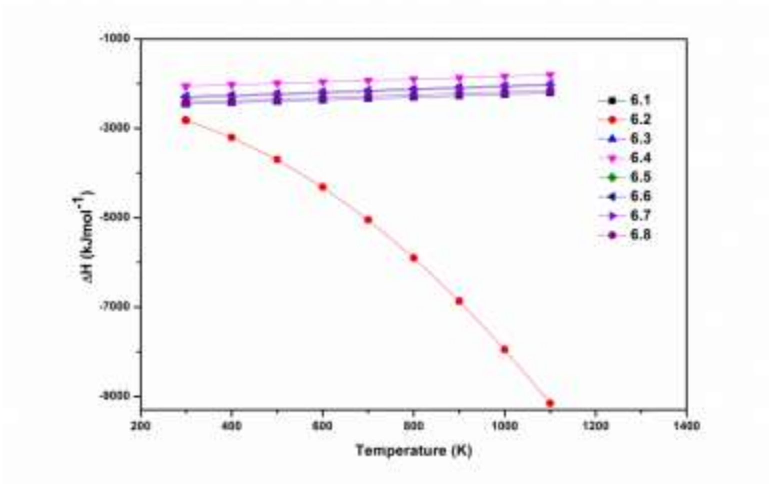

Fig. S 6 Variation ~~with~~ in heat of formation with temperature for reduction of MoO_3+Mo_4O_{11} to MoO_2.

Appendix S 7

$MoO_3 + Mo_4O_{11} + 7H_2 + 6C \rightarrow 5/2Mo_2C + 3.5CO_2 + 7H_2O$ (S 7.1)

$MoO_3 + Mo_4O_{11} + 9H_2 + 10C \rightarrow 5/2Mo_2C + 6CO + 1/2CH_4 + 8H_2O + C$ (S 7.2)

$MoO_3 + Mo_4O_{11} + 6CH_4 \rightarrow 5/2Mo_2C + 5/2CO_2 + 9H_2O + 3H_2 + C$ (S 7.3)

$MoO_3 + Mo_4O_{11} + 6CH_4 \rightarrow 5/2Mo_2C + 5/2CO_2 + 9H_2O + CH_4 + H_2$ (S 7.4)

$MoO_3 + Mo_4O_{11} + 8CH_4 + H_2 \rightarrow 5/2Mo_2C + 1/2CO_2 + 10H_2O + 3CO + 2CH_4 + 3H_2$ (S 7.5)

$MoO_3 + Mo_4O_{11} + 6CH_4 + H_2 \rightarrow 5/2Mo_2C + 1/2CO_2 + 11H_2O + 2CO + C + 2H_2$ (S 7.6)

$MoO_3 + Mo_4O_{11} + 5C_2H_4 \rightarrow 5/2Mo_2C + 3/2CO_2 + 7H_2O + 4C + 4H_2 + 2CO_2$ (S 7.7)

$MoO_3 + Mo_4O_{11} + 4C_2H_6 \rightarrow 5/2Mo_2C + 3/2CO_2 + 9H_2O + 2CO + 3H_2 + 2C$ (S 7.8)

$MoO_3 + Mo_4O_{11} + 5C_2H_4 \rightarrow 5/2Mo_2C + 3/2CO_2 + 6H_2O + 5CO + CH_4 + 2H_2$ (S 7.9)

$MoO_3 + Mo_4O_{11} + 3C_3H_6 \rightarrow 5/2Mo_2C + 1/2CO_2 + 6H_2O + 5CO + 3H_2 + CO_2$ (S 7.10)

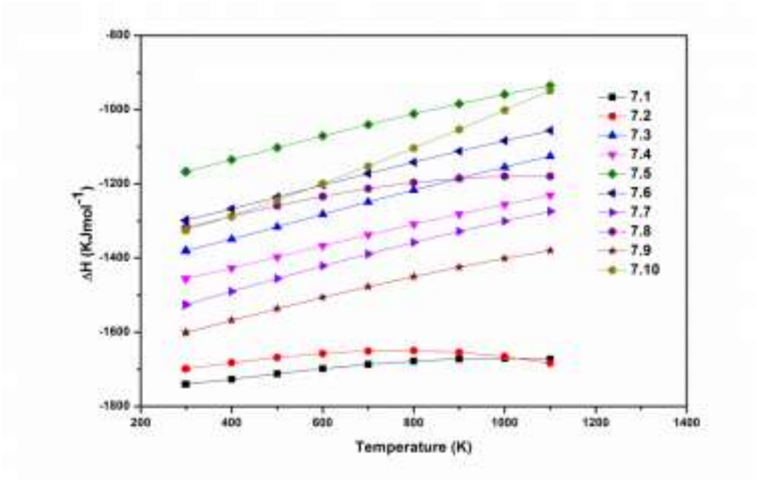

Fig. S 7 Variation ~~with~~ in heat of formation with temperature for reduction of MoO_3+Mo_4O_{11} to MoO_2 calculated for above reactions at different temperatures up to 800 °C (1073K).

Appendix S 8

Kinetic Theory

The rate of solid state thermal decomposition of chemical reaction can be written as [6]:

$$\frac{d\alpha}{dt} = kf(\alpha) \qquad (\boldsymbol{S\ 8.1})$$

$$\alpha = \frac{m_0 - m_t}{m_0 - m_f}$$

where n is the order of reaction, α is the degree of conversion, m_o is the initial mass, m_t is the mass at time t, m_f is the final mass and k is the rate constant which can be expressed by Arrehenius equation:

$$k = Ae^{-\frac{E_a}{RT}} \qquad (\boldsymbol{S\ 8.2})$$

where, A is the pre-exponential factor, E_a is the activation energy, R is the gas constant (8.314 J K^{-1} mol^{-1}) and T is the absolute temperature. The final equation for rate of thermal decomposition process can be expressed as:

$$\frac{d\alpha}{dt} = Ae^{-\frac{E_a}{RT}}f(\alpha) \qquad (\boldsymbol{S\ 8.3})$$

If the temperature increases in a dynamic TG experiment at linear heating rate $\beta = \frac{dT}{dt}$, the above equation can be written as:

$$\int_0^\alpha \frac{d\alpha}{f(\alpha)} = \frac{A}{\beta}\int_0^T e^{-E_{a/RT}}\, dT = g(\alpha) \qquad (\boldsymbol{S\ 8.4})$$

where, $g(\alpha)$ is the integral function of degree of conversion. This equation serves as a base for various kinetic methods used to determine thermal kinetic parameters.

Coats-Redfern method

This method is extensively used to determine the reaction mechanism involved in the thermal decomposition of the sample [28]. Coats and Redfern method is an integral method based on Eq. (4) and can be expressed as:

$$ln\left(\frac{g(\alpha)}{T^2}\right) = ln\left(\frac{AR}{\beta E_a}\right) - \frac{E_a}{RT} \quad \textbf{(S 8.5)}$$

A plot between $ln\left(\frac{g(\alpha)}{T^2}\right)$ and $\frac{1000}{T}$ provides information regarding the kinetic parameters of a reaction. The activation energy (E_a) is determined from the slope, whereas the value of pre-exponential factor (A) is calculated from the intercept. However, it is essential to calculate the accurate value of $g(\alpha)$ which can only be obtained by employing various reaction mechanism involved in a thermal decomposition process, as listed in Table S1 [7]. The choice of exact $g(\alpha)$ depends on the value of correlation factor (R^2).

References

[1] Vives, S., Gaffet, E. & Meunier, C. X-ray diffraction line profile analysis of iron milled powders. *Mater. Sci. Eng. A* **366,** 229-238 (2004).

[2] Rosenberg, Y., Machavariant, V. S., voronel, A., Garber, S., Rubshtein, A., Frenkel, A. I. & Stern, E. A. Strain energy density in the x-ray powder diffraction from mixed crystals and alloys. *J. Phys. Condens. Mater.* **12**, 8081-8088 (2000).

[3] Liu, H., Zhu, J., Lai, Z., Zhao, R. & He, D. A first principle study on structural and electronic properties of Mo_2C. *Scripta Mater.* **60**, 949-952 (2009).

[4] Zhang, J. M., Zhang, Y., Xu, K. W. & Ji, V. General compliance transformation relation and applications for anisotropic hexagonal metals. *J. Solid Stat. Commun.* **139**, 87-91(2006).

[5] Liu,Y. Z., Jiang, Y. H., Liu, X. F., Feng, J. Elastic and thermodynamic properties of Mo_2C polymorphs from first principles calculations. *Ceram. Int.* **41**, 5329-5246 (2015).

[6] Jain, A. A., Mehra, A. Ranade, V. V. Processing of TGA data: Analysis of isoconversional and model fitting methods. *Fuel* **165**, 490–498(2016).

[7] Núñez, L., Fraga, F., Núñez, M. R. & Villanueva, M. Thermogravimetric study of the decomposition process of the system BADGE (n = 0)/1,2 DCH. *Polymer* **41,** 4635–4641 (2000).
